# Vitamin D_3_ suppresses morphological evolution of the cribriform cancerous phenotype

**DOI:** 10.18632/oncotarget.8863

**Published:** 2016-04-20

**Authors:** Ravi K. Deevi, Jane McClements, Karen D. McCloskey, Aliya Fatehullah, Dorota Tkocz, Arman Javadi, Robyn Higginson, Victoria Marsh Durban, Marnix Jansen, Alan Clarke, Maurice B. Loughrey, Frederick C. Campbell

**Affiliations:** ^1^ Centre for Cancer Research and Cell Biology, Queen's University of Belfast, Belfast, UK; ^2^ Northern Ireland Molecular Pathology Laboratory, Centre for Cancer Research and Cell Biology, Queens University Belfast and Belfast Health and Social Care Trust, Belfast, UK; ^3^ European Cancer Stem Cell Research Institute, Cardiff University, Cardiff, UK; ^4^ Barts Cancer Institute, Queen Mary University of London, John Vane Science Centre, London, UK

**Keywords:** colorectal cancer, molecular oncology, vitamin D receptor gene, morphology, prognosis

## Abstract

Development of cribriform morphology (CM) heralds malignant change in human colon but lack of mechanistic understanding hampers preventive therapy. This study investigated CM pathobiology in three-dimensional (3D) Caco-2 culture models of colorectal glandular architecture, assessed translational relevance and tested effects of 1,25(OH)_2_D_3_, the active form of vitamin D. CM evolution was driven by oncogenic perturbation of the apical polarity (AP) complex comprising *PTEN, CDC42* and *PRKCZ* (phosphatase and tensin homolog, cell division cycle 42 and protein kinase C zeta). Suppression of AP genes initiated a spatiotemporal cascade of mitotic spindle misorientation, apical membrane misalignment and aberrant epithelial configuration. Collectively, these events promoted “Swiss cheese-like” cribriform morphology (CM) comprising multiple abnormal “back to back” lumens surrounded by atypical stratified epithelium, in 3D colorectal gland models. Intestinal cancer driven purely by *PTEN*-deficiency in transgenic mice developed CM and in human CRC, CM associated with *PTEN* and *PRKCZ* readouts. Treatment of *PTEN*-deficient 3D cultures with 1,25(OH)_2_D_3_ upregulated *PTEN*, rapidly activated *CDC42* and *PRKCZ*, corrected mitotic spindle alignment and suppressed CM development. Conversely, mutationally-activated *KRAS* blocked 1,25(OH)_2_D_3_ rescue of glandular architecture. We conclude that 1,25(OH)_2_D_3_ upregulates AP signalling to reverse CM in a *KRAS* wild type (wt), clinically predictive CRC model system. Vitamin D could be developed as therapy to suppress inception or progression of a subset of colorectal tumors.

## INTRODUCTION

Oncogenic perturbation of cell-cell interactions and hierarchical, three-dimensional (3D) tissue organization characterizes cancer development [1] and progression [2]. Cribriform morphology (CM) is commonly viewed as a histopathological correlate of malignant transformation in human colon [3], detectable in malignant polyps [4] and early invasive colorectal cancer (CRC) [5]. CM has a “Swiss-cheese - like” histological appearance, characterized by multiple abnormal lumens surrounded by stratified malignant epithelium [6]. While CM pathobiology remains unclear, lumen formation and epithelial configuration are governed by mitotic spindle orientation [7, 8].

Spindle alignment is controlled by the apical polarity complex including *PTEN*, *CDC42, PRKC* and *PARD* genes [9, 10]. *PTEN* is a tumor suppressor that coordinates the *CDC42-PRKCZ-PARD* complex [11, 12] and regulates spindle orientation in nonpolarized cultured cells [13]. *PRKCZ* spatially regulates *PARD3* that cooperates with the heterotrimeric G protein subunit *GNAI3* (guanine nucleotide binding protein alpha inhibiting activity polypeptide 3; also known as Gαi3) to localize the spindle orientation protein, G-protein signalling modulator *2 (GPSM2;* also known as LGN*)* [14]. *PARD3* directs the orientation of pulling forces linked through *GPSM2* to spindle microtubules for appropriate spindle alignment [14]. Perturbation of this machinery drives transition to dysplasia in Drosophila [15] but effects on colorectal glandular architecture remain unclear.

Components of the apical polarity complex including *PTEN* [16] and *PRKCZ* [17] can be enhanced by vitamin D (Vit-D) treatment. This secosteroid also promotes rapid calcium (Ca^2+^) signalling [18] that activates *CDC42* [19, 20] and controls spindle microtubule dynamics [21]. Vit-D influences molecular to multicellular scales of tissue organization [22-24] and suppresses CRC progression [25, 26]. Conversely, mutationally-activated *KRAS* may inhibit Vit-D growth control [27, 28] by unclear mechanisms.

In this study, we investigated CM pathobiology using three-dimensional (3D) organotypic CRC culture model systems. We tested 1,25(OH)_2_D_3_ treatment and investigated effects of mutationally-activated *KRAS*. To investigate translational relevance of our experimental findings, we conducted histologic, immunohistochemical and/or RNA *in situ* hybridization assays in murine and human tumors.

## RESULTS

### *PTEN* deficiency induces mitotic spindle misorientation, epithelial stratification and cribriform morphology

**Figure 1:**
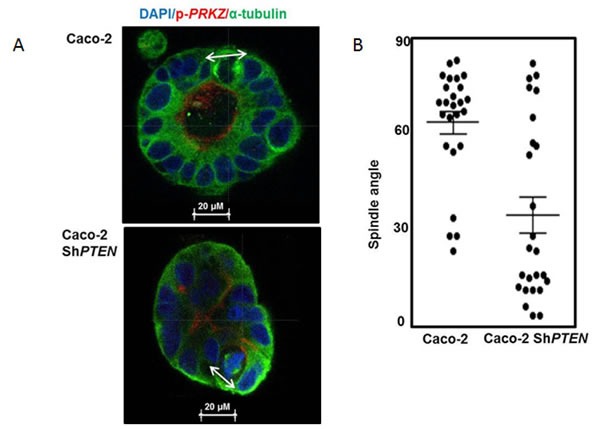
**A.** *PTEN* knockdown misorientates the mitotic spindle. Caco-2 and Caco-2 Sh*PTEN* gland cultures at 4 days. DAPI (blue), p-*PRKCZ* (red) and anti-α -tubulin (green) were used as markers of nuclear DNA, apical *PRKCZ* activity and spindle microtubules respectively. Mitotic spindle orientation (double headed solid white arrow) is positioned approximately perpendicular to the Caco-2 gland lumen but is misorientated in Caco-2 Sh*PTEN* glands. Scale bar 20 μm. **B**. Summary angles between spindle midpoints and gland lumens. Caco-2 - 65.5 ± 3.7^0^ *vs* Caco-2 Sh*PTEN* - 34.9 ± 5.9^0^; (*p* < 0.01; ANOVA). **C**. CM evolution in Caco-2 Sh*PTEN* glands. At (i) 4 days, the mitotic spindle (anti-α-tubulin; green) is misorientated relative to gland centres (double headed white arrow), accompanied by misalignment of the apical membrane (AM; red; fine interrupted white arrows). At (ii) 8 days, secretion-driven expansion of ectopic AM forms multiple abnormal lumens (solid white arrows), accompanied by epithelial stratification (broad interrupted white arrows). These phenomena induce well-formed cribriform morphology at (iii) 12 days, characterized by multiple gland lumens surrounded by stratified epithelium (broad interrupted white arrows). Scale bar 20 μm. These glandular architecture alterations are evocative of cribriform morphology in human CRC (iv; H&E section of human CRC showing a glandular structure with multiple aberrant lumens, surrounded by abnormal stratified epithelium [broad interrupted white arrows]). **D**. Schematic of spindle orientation and epithelial configuration. During mitosis, the plane of cell cleavage (double headed black arrow - interrupted line) lies perpendicular to the spindle midpoint. The mitotic spindle is orientated (MSO) (green microtubules/black centrosomes) approximately perpendicular (┴) to the cell long axis. With this configuration, cell division generates an epithelial monolayer within glands, with cells linked by apical junctions (orange). Apical membranes (AM; red) face a central lumen. When mitotic spindle orientation (MSO) is parallel (═) to the cell long axis cell division generates stratified epithelium (light brown). **E**. Schematic of CM evolution. Spindle misorientation (i) induces epithelial stratification (light brown) and AM (red) misalignment. (ii) Secretion driven expansion of ectopic AM leads to multilumen formation [7]. (iii) Collectively, these phenomena induce CM (iv). **F**. Summary effects of ***PTEN*** knockdown on epithelial stratification. Values shown indicate % glands with any stratification (Caco-2 - 22.7 ± 7.5% *vs* Caco-2ShPTEN - 71.3 ± 12.6%; *p* < 0.03; ANOVA). **G**. ***PTEN*** knockdown suppresses ***CDC42/PRKCZ*** and promotes ***PARD3*** mislocalization. *CDC42*-GTP, apical p-*PRKCZ* immunofluorescence and *PARD3* localization are shown. *PARD3* localizes to apical junction regions in Caco-2 glands. In Caco-2 Sh*PTEN* glands low *CDC42* activity, low apical p-*PRKCZ* and *PARD3* mislocalization associate with CM. Multilumen formation indicated by solid white arrows. Scale bar 20 μm. **H**. Summary effects of PTEN knockdown on *CDC42* and ***p-****PRKCZ*. Values represent fold differences in *CDC42-GTP* and apical p-*PRKCZ* intensities respectively in Caco-2 Sh*PTEN vs* Caco-2 glands; *CDC42-GTP -* 0.40 ± 0.03; p-*PRKCZ* - 0.45 ± 0.04; *p* < 0.01 ANOVA).

The tumor suppressor *PTEN* regulates *CDC42* and apical *PRKCZ* activity [11, 12] that have a mechanistic role in spindle orientation, lumen formation and 3D epithelial morphology [7, 9, 29]. Downstream of *PTEN, CDC42* promotes recruitment and activation of *PRKCZ* at the apical domain that localizes *PARD3* [30] to a nascent apical junctional complex required for spindle alignment [14]. Here we show that *PTEN-*deficiency induces spindle misorientation (Figure 1A, 1B), epithelial stratification and multilumen formation in Caco-2 Sh*PTEN* glandular structures [glands] (Figure 1C [i-iii]) consistent with CRC cribriform morphology [CM] (Figure 1C [iv]). Epithelial stratification was typically focal in early developing glands, becoming organised around multiple abnormal lumens at later stages (Figure 1C [i-iii]). Focal stratification without multilumen formation was observed in some late stage Caco-2 Sh*PTEN* glands (Supplementary Figure S1A). Schematics for epithelial stratification and cribriform morphogenesis are shown (Figure 1D, 1E). Epithelial stratification in Caco-2 and Caco-2 Sh*PTEN* glands is summarised in Figure 1F.

Functional readout of *PRKCZ* activity at the apical domain can be provided by signal intensity of apical phospho-*PRKCZ (*p-*PRKCZ)* [9] or *SLC9A3R1* [Solute carrier family 9, subfamily A (NHE3, cation proton antiporter 3), member 3 regulator 1; also known as NHERF-1 (Na^+^/H^+^ exchange regulatory factor 1)] [12]. In accord with *PTEN* regulation of the *CDC42/PRKCZ/PARD* complex, we show low *CDC42*-GTP (guanine triphosphate) immunofluorescence, reduced apical p-*PRKCZ* signal intensity (Figure 1G, 1H) and displacement of *PARD3* from the subapical domain in *PTEN*-deficient Caco-2 Sh*PTEN* glands (Figure 1G). Caco-2 sh*PTEN* glands were more cellular (Supplementary Figure S1B) with greater maximum surface area (Supplementary Figure S1C) than Caco-2 glands, consistent with impairment of *PTEN* antiproliferative activity. SiRNA knockdown of *PTEN* in SK-CO-15 CRC cells (Supplementary Figure S1D) also induced cribriform architecture with multiple aberrant lumens (Supplementary Figure S1E, S1F) and increased cellularity, in 3D cultures (Supplementary Figure S1G).

### 1,25(OH)_2_D_3_ upregulates *PTEN/CDC42/ PRKCZ* signalling to control gland morphology

**Figure 2:**
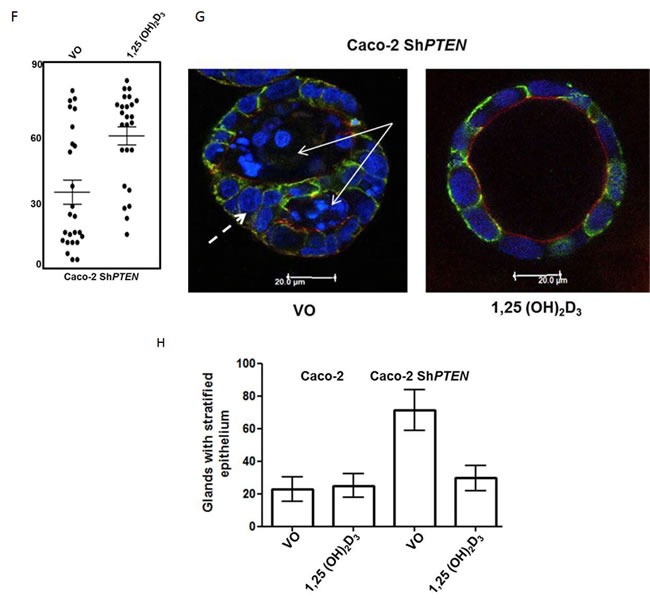
**A.** Effects of 1,25(OH)_2_D_3_ treatment on ***VDR*** expression. (i) Treatment effects on vitamin D receptor (*VDR*) expression in Caco-2 and Caco-2 Sh*PTEN* cells. (ii) Summary densitometry values represent fold expression changes relative to VO treated Caco-2 cells (Caco-2 1,25(OH)_2_D_3_ - 1.47 ± 0.04; Caco-2 Sh*PTEN* VO - 0.68 ± 0.04; Caco-2 Sh*PTEN* 1,25(OH)_2_D_3_ 1.40 ± 0.6; *p* < 0.01; ANOVA). **B**. Effects of 1,25(OH)_2_D_3_ treatment on *PTEN* and ***CDC42-***GTP. (i) Treatment effects on *CDC42*-GTP and *PTEN* in Caco-2 and Caco-2 Sh*PTEN* cells. Summary densitometry values for (ii) *CDC42*-GTP and (iii) *PTEN* represent fold expression changes relative to VO treated Caco-2 cells (*CDC42*-GTP - Caco-2 1,25(OH)_2_D_3_ - 2.0 ± 0.14;Caco-2 Sh*PTEN* VO - 0.68 ± 0.04; Caco-2 Sh*PTEN*; 1,25(OH)_2_D_3_ - 1.50 ± 0.06; Caco-2 1,25(OH)_2_D_3_ - 1.67 ± 0.09; Caco-2 Sh*PTEN* VO - 0.47 ± 0.044; Caco-2 Sh*PTEN* 1,25(OH)_2_D_3_ - 1.3 ± 0.06; *p* < 0.01; ANOVA). **C**. 1,25(OH)_2_D_3_ treatment upregulates apical polarity signalling (p-*PRKCZ* and *SLC9A3R1*). Apical p-*PRKCZ* (red) and *SLC9C3R1* (green) signal intensities in Caco-2 glands after treatment (VO - top row *vs* 1,25(OH)_2_D_3_ - bottom row). Scale bar 20 μm. **D**. Summary treatment effects on (i) apical *p-PRKCZ* and (ii) *SLC9A3R1*. Values represent fold changes of apical p-*PRKCZ* (3.3 ± 0.37) and *SLC9A3R1* (2.29 ± 0.18) signal intensities after 1,25(OH)_2_D_3_ *vs* VO control; *p* < 0.01; ANOVA). **E**. 1,25(OH)_2_D_3_ treatment restores spindle orientation in Caco-2 *ShPTEN* glands. Treatment by vehicle only (VO; top panel) or 1,25(OH)_2_D_3_ (bottom panel). Imaging by DAPI (blue) and anti-α-tubulin (green). Spindle orientation indicated by double-headed white arrows in Merge images. Scale bar 20 μm. **F**. Summary effects of 1,25(OH)_2_D_3_ treatment on spindle orientation. Summary spindle angles in Caco-2-Sh*PTEN* glands after treatment (VO - 35 ± 5.9^0^ *vs* 1,25(OH)_2_D_3_ - 63 ± 4.5^0^; *p* < 0.01; ANOVA). **G**. 1,25(OH)_2_D_3_ treatment suppresses development of cribriform morphology. Epithelial stratification (broad interrupted white arrow) and multiple lumens (solid white arrows) in Caco-2Sh*PTEN* glands after treatment by VO *vs* 1,25(OH)_2_D_3._ Scale bar 20 μm. **H**. 1,25(OH)_2_D_3_ treatment suppresses epithelial stratification. Caco-2 - VO - 22.6 ± 7.5%; 1,25(OH)_2_D_3_ - 25.0 ± 7.0%; Caco-2 Sh*PTEN* - VO - 71.3 ± 12.6%; 1,25(OH)_2_D_3_ - 29.6 ± 7.6%; Two way ANOVA - *p* < 0.02 for effects of cell type and *p* < 0.04 for cell type-treatment interaction.

1,25(OH)_2_D_3_ treatment enhanced vitamin D receptor (*VDR)* expression, perinuclear and nuclear *VDR* localization in Caco-2 and Caco-2Sh*PTEN* cells, indicating biological responsiveness of the model system (Figure 2A; Supplementary Figure S2A, S2B). Treatment also increased *PTEN* expression (Figure 2B) and activation of *CDC42* (Figure 2B). 1,25(OH)_2_D_3_ treatment enhanced apical p-*PRKCZ* and *SLC9A3R1* signal intensities in Caco-2 glands (Figure 2C, 2D) and restored spindle orientation (Figure 2E, 2F), formation of epithelial monolayers within glands (Figure 2G, 2H) and single lumen formation at progressive stages of Caco-2 Sh*PTEN* gland development (Supplementary Figure S2C, S2D). Continuous 1,25(OH)_2_D_3_ treatment sustained long term (20 days) rescue of Caco-2 Sh*PTEN* gland morphology (Supplementary Figure S2E, S2F) while cessation of treatment at 4 days induced reversal to CM (Supplementary Figure S2G, S2H). 1,25(OH)_2_D_3_ treatment also suppressed Caco-2 Sh*PTEN* gland cellularity (Supplementary Figure S2I). Hence, 1,25(OH)_2_D_3_ targets spindle regulatory machinery to control 3D colorectal gland morphology.

### 1,25(OH)_2_D_3_ rescues defective morphology of *PTEN*-deficient glands by targeting *CDC42/PRKCZ* crosstalk

**Figure 3:**
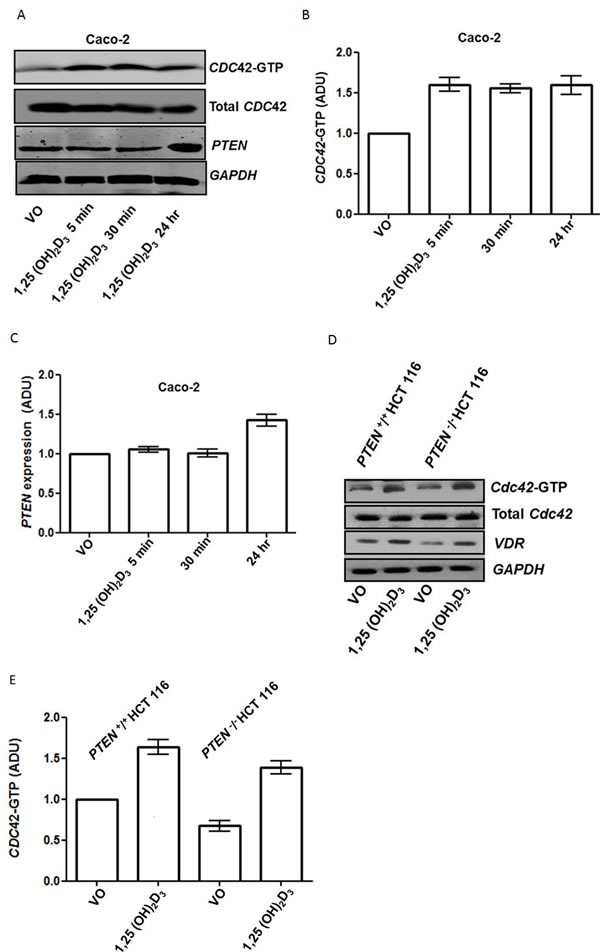
**A.** Timecourse of 1,25(OH)_2_D_3_ modulation of ***CDC42***-GTP and ***PTEN***. *GAPDH* loading control. **B**. Summary effects of 1,25(OH)_2_D_3_ on *CDC42*-GTP. Densitometry values are fold differences of *CDC42*-GTP levels in Caco-2 cells over VO control (1,25(OH)_2_D_3_ - 5 mins - 1.6 ± 0.09; 30 mins - 1.55 ± 0.05; 24h - 1.59 ± 0.12; p<0.01; ANOVA). **C**. Summary effects of 1,25(OH)_2_D_3_ on ***PTEN***. Densitometry values are fold differences of *PTEN* levels in Caco-2 cells over VO control (1,25(OH)_2_D_3_ 5 mins - 1.05 ± 0.03; 30 mins - 1.0 ± 0.05; 24h - 1.46 ± 0.07; p<0.01 for 24hr; ANOVA). **D**. 1,25(OH)_2_D_3_ activation of ***CDC42*** is ***PTEN****-*independent. Effects of 1,25(OH)_2_D_3_ treatment on *CDC42*-GTP levels in *PTEN*^+/+^ and *PTEN*^−/−^ HCT116 cells. *VDR* indicates biological responsiveness and *GAPDH* used as loading control. **E**. Summary effects of 1,25(OH)_2_D_3_ on ***CDC42***-GTP in ***PTEN***^+/+^ and ***PTEN***^−/−^ HCT116 cells. Values shown are fold differences over VO control (*PTEN* ^+/+^ HCT116 1,25(OH)_2_D_3_ - 1.63 ± 0.09; *PTEN* ^−/−^ HCT116 VO - 0.67 ± 0.07; *PTEN* ^−/−^ HCT116 1,25(OH)_2_D_3_ - 1.41 ± 0.04; p<0.01 ANOVA). **F**. Inhibition of *PRKCZ* suppresses 1,25(OH)_2_D_3_ rescue of Caco-2 Sh***PTEN*** gland morphology. Apical p-*PRKCZ* and *CTNNB1* were used as apical and basolateral membrane markers respectively. Multilumen formation indicated by white solid arrows in Merge images. Top row - VO; Second row −1,25(OH)_2_D_3_; Third row *PRKCZ* pseudosubstrate inhibitor (*PRKCZ*I); Bottom row combined 1,25(OH)_2_D_3_/*PRKCZ*I treatment. Scale bar 20 μm. **G**. Summary treatment effects on single lumen formation in Caco-2 Sh***PTEN*** glands. Values shown are fold differences over VO control (1,25(OH)_2_D_3_ - 2.01 ± 0.1_;_ *PRKCZ*I - 0.59 ± 0.07; 1,25(OH)_2_D_3_/*PRKCZ*I - 0.84 ± 0.07; p<0.01 for 1,25(OH)_2_D_3_ *vs* VO; ANOVA).

1,25(OH)_2_D_3_ initiates rapid nongenomic biological responses, in addition to transcriptional effects on target genes [31]. To identify principal 1,25(OH)_2_D_3_ - responsive effectors within the *PTEN/CDC42/PRKCZ* complex, we conducted timescale, transfection and treatment studies. We found that 1,25(OH)_2_D_3_ treatment activated *CDC42* within 5 minutes but only upregulated *PTEN* by 24 hrs (Figure 3A-3D). Furthermore, we showed that 1,25(OH)_2_D_3_ upregulated *CDC42*-GTP in both *PTEN* ^+/+^ and *PTEN* ^−/−^ HCT116 cells (Figure 3D, 3E). Hence, 1,25(OH)_2_D_3_ upregulates *PTEN* but can also activate *CDC42* by *PTEN*-independent mechanisms. To disrupt morphogenesis of Caco-2 glands, we stably transfected cells with dominant negative (DN) *CDC42* (Supplementary Figure S3A, S3B) or treated cultures with a *PRKCZ* pseudosubstrate inhibitor (*PRKCZ*I) (Supplementary Figure S3C, S3D). Aberrant gland morphology resulting from DN *CDC42* transfection or *PRKCZ*I treatment could not be reversed by 1,25(OH)_2_D_3_ treatment (Supplementary Figure S3A-S3D). Furthermore, rescue of Caco-2 Sh*PTEN* gland morphology by 1,25(OH)_2_D_3_ treatment was blocked by *PRKCZ*I treatment (Figure 3F, 3G). These findings show that 1,25(OH)_2_D_3_ can rescue aberrant morphology of Caco-2 Sh*PTEN* glands by targeting *CDC42/PRKCZ* crosstalk.

### 1,25(OH)_2_D_3_ activates *CDC42/PRKCZ* signalling through Ca^2+^ flux

**Figure 4:**
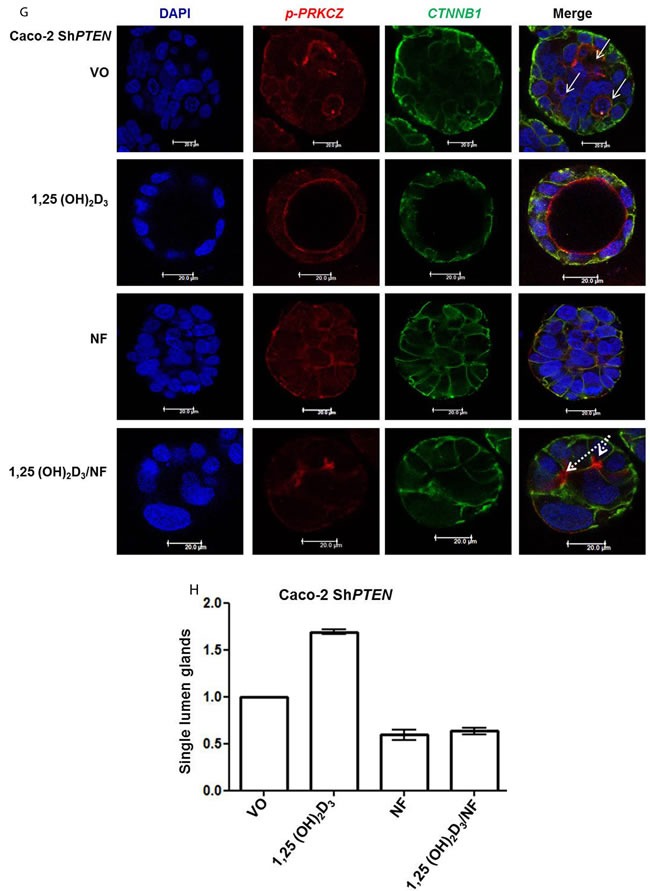
**A.** Expression of L-type voltage-dependent Ca^2+^ channels (LTVDCC) in Caco-2 glands. DAPI, p-*PRKCZ* and Cav 1.3 were used as markers of nuclear DNA, apical *PRKCZ* activity and the β-subunit of the L-type voltage-dependent Ca^2+^ channel, respectively. Expression of the β-subunit of LTVDCCs is predominantly basolateral in polarized epithelium [46]. **B**. 1,25 (OH)_2_D_3_ promotes Ca^2+^ flux I. Representative fluorescence images of Caco-2 cells loaded with fluo-4AM before (i) and after (ii) exposure to 1,25 (OH)_2_D_3_ (100nM). An increase in intracellular Ca^2+^ is shown by an increase in fluorescence intensity.II. Intensity-time plot of the effect of 1,25 (OH)_2_D_3_.III. Summary bar chart of fluorescence amplitude evoked by 1,25 (OH)_2_D_3_ (*n* = 13 cells) or the Ca^2+^-ionophore, ionomycin (1μM, *n* = 27 cells; positive control). **C**. Calcium channel blockade impedes morphogenesis. (i) Caco-2 gland morphogenesis at 4 days after VO (top row), NF (second row) or 1,25(OH)_2_D_3_/NF combined treatment (bottom row). Multiple lumen formation indicated by solid white arrows in Merge images. Scale bar - 20 μM.(ii) Summary values represent fold change of single lumen formation relative to VO control (NF - 0.60 ± 0.06; 1,25(OH)_2_D_3_/NF in combination - 0.70 ± 0.054; *p* < 0.01 ANOVA). **D**. CaM-KII inhibition impedes morphogenesis. (i) Caco-2 gland morphogenesis at 4 days after VO (top row), KN-93 (second row) or 1,25(OH)_2_D_3_/KN-93 combined treatment (bottom row). Aberrant lumens or ectopic AM foci (indicated by solid or fine interrupted white arrows respectively in Merge). Scale bar - 20 μM. (ii) Summary values represent single lumen formation relative to VO control (KN-93 - 0.50 ± 0.06; 1,25(OH)_2_D_3_/KN-93 - 0.60 ± 0.062; *p* < 0.01 ANOVA). **E**. ***VDR*** knockdown suppresses 1,25(OH)_2_D_3_ rescue of morphogenesis. Images show Caco-2 Sh*PTEN* gland morphogenesis after SiRNA transfection and treatment, at 4 days of culture. Top 2 panels show effects of VO or 1,25(OH)_2_D_3_ treatment combined with non-targeting (NT) SiRNA; bottom 2 panels - VO or 1,25(OH)_2_D_3_ treatment combined with *VDR* SiRNA. DAPI, p*-PRKCZ* and *VDR* imaging. Ectopic AM foci indicated by fine interrupted white arrows in Merge images. Scale bar 20 μM. **F**. Summary effects of ***VDR*** knockdown on 1,25(OH)_2_D_3_ rescue of Caco-2 Sh***PTEN*** gland morphogenesis. Values shown represent fold differences of single lumen formation against Caco-2 Sh*PTEN* glands treated by VO and transfected by NT SiRNA. NT SiRNA + 1,25(OH)_2_D_3_ - 1.77 ± 0.09; VDR SiRNA +VO = 0.51 ± 0.04; VDR SiRNA + 1,25(OH)_2_D_3_ = 0.6 ± 0.06; *p* < 0.01; ANOVA. **G.** Calcium channel blockade impedes 1,25(OH)_2_D_3_ rescue of Caco-2 Sh*PTEN* gland morphogenesis. Treatments were VO (top panel), 1,25(OH)_2_D_3_ (second panel), NF (third panel), NF/1,25(OH)_2_D_3_ (fourth panel). Multiple lumens and ectopic AM without lumens indicated by solid and fine interrupted white arrows respectively in Merge images. Scale bar 20 μM. **H**. Summary treatment effects on single lumen formation in Caco-2 Sh***PTEN*** glands. Values are expressed as fold changes over VO control (1,25(OH)_2_D_3_ - 1.69 ± 0.03_;_ NF - 0.59 ± 0.05; 1,25(OH)_2_D_3_/NF - 0.63 ± 0.03; *p* < 0.01 ANOVA).

1,25(OH)_2_D_3_ promotes rapid *VDR*-dependent Ca^2+^ flux [18], mediated through L-type voltage-dependent calcium channels (LTVDCCs) [32] and CaM-KII activity [32]. Ca^2+^ flux and/or calcium-calmodulin dependent protein kinase (CaM-KII) activity can enhance *CDC42*-GTP polarity signalling [19, 20]. In this study, Caco-2 glands expressed LTVDCCs predominantly at basolateral membranes (Figure 4A). 1,25(OH)_2_D_3_ treatment increased intracellular Ca^2+^ concentration in Caco-2 cells (Figure 4BI, 4BII) and was compared against the Ca^2+^-ionophore, ionomycin as positive control (Figure 4BIII). SiRNA knockdown of *VDR* inhibited 1,25(OH)_2_D_3_-mediated *CDC42* activation (Supplementary Figure S4A). Treatment of cells with the LTVDCC inhibitor nifedipine (NF) or the CaM-KII inhibitor KN-93 also suppressed 1,25(OH)_2_D_3_-mediated *CDC42* activation but did not affect *PTEN* levels (Supplementary Figure S4B, S4C). Treatment of developing Caco-2 glands with NF or KN-93 suppressed p-*PRKCZ* enrichment at apical domains, induced apical membrane misalignment and formation of multiple poorly formed lumens. These phenotypes in Caco-2 glands were not rescued by 1,25(OH)_2_D_3_ treatment (Figure 4C, 4D). Furthermore, 1,25(OH)_2_D_3_ rescue of morphogenesis in Caco-2 Sh*PTEN* glands was blocked by transfection with *VDR* SiRNA (Figure 4E, 4F) or cotreatment with NF (Figure 4G, 4H) or KN-93 (Supplementary Figure S4D, S4E). Taken together, these findings implicate *VDR* and Ca^2+^/CaM-KII signalling in 1,25(OH)_2_D_3_-mediated upregulation of *CDC42/PRKCZ* and reversal of cribriform morphology in *PTEN*-deficient colorectal glands.

### Mutationally-activated *KRAS* suppresses 1,25(OH)_2_D_3_ rescue of gland morphology

**Figure 5:**
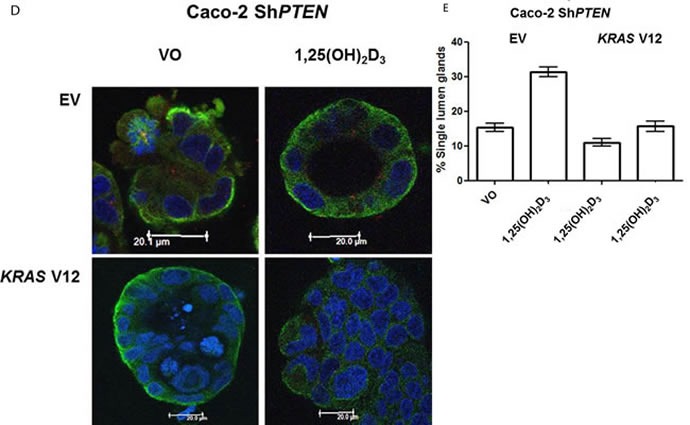
**A.** *KRASV12* impedes morphogenesis. Caco-2 (top 2 rows) or Caco-2 Sh*PTEN* glands (bottom 2 rows) were transfected with empty vector (EV) or mutationally-activated *KRAS* (*KRAS V12*) and imaged for DAPI (blue), *KRAS* (green) and phospho-*PRKCZ (red). KRAS V12* transfections in both cell types induced increased *KRAS* immunoreactivity and perturbed gland morphology, leading to solid cell-filled glands lacking a central lumen. Multilumen formation is observed in EV transfected Caco-2 Sh*PTEN* glands, although this phenotype is suppressed by *KRAS V12* transfection. **B**. Summary effects of ***KRAS V12*** transfection on gland morphogenesis. Values shown represent % single lumen formation in Caco-2 *vs* Caco-2Sh*PTEN* glands after EV or *KRAS V12* transfection at 4 days of culture (Caco-2 - 24.6 ± 0.88 (EV) *vs* 13.6 ± 1.2 (*KRAS*V12); Caco-2 Sh*PTEN* −15.3 ± 1.3 (EV) *vs* 11.0 ± 1.5 (*KRAS*V12; *p* < 0.01;ANOVA). **C**. Summary effects of ***KRAS V12*** transfection on apical p-***PRKCZ*** intensity in Caco-2 glands. Results shown represent fold differences against EV transfected Caco-2 glands. *KRAS* V12 transfectants = 0.495 ± 0.1; *p* < 0.01; Student's t test. **D**. ***KRAS V12*** suppresses 1,25(OH)_2_D_3_ rescue of gland morphology. Top panel - transfection by empty vector (EV) only and treatment by VO or 1,25(OH)_2_D_3._ Bottom panel - transfection with *KRAS*V12, combined with VO or 1,25(OH)_2_D_3_ treatment. **E**. Summary effects of ***KRAS*** V12 ***vs*** EV transfection on 1,25(OH)_2_D_3_ rescue of Caco-2 Sh***PTEN*** gland morphology. Values shown represent single lumen formation after transfection and treatment at 4 days of culture, namely EV/VO - 15.3 ± 1.2 (VO); EV/1,25(OH)_2_D_3_ - 31.3 ± 1.46; *KRAS*/VO - 11.0 ± 1.15; *KRAS*/1,25(OH)_2_D_3 -_ 15.67 ± 1.45; *p* < 0.001; Two Way ANOVA).

Because *KRAS* can suppress 1,25(OH)_2_D_3_ growth control [27], we investigated effects of mutationally-activated *KRAS* V12 in our 3D Caco-2 and Caco-2 Sh*PTEN* model systems. Transfection with *KRAS* V12 inhibited lumen formation to yield glands that were cell-filled and solid in appearance (Figure 5A, 5B), in accord with previous findings [33]. Furthermore, *KRAS* V12 transfection suppressed apical localization of *PRKCZ* in Caco-2 glands (Figure 5A, 5C) and inhibited 1,25(OH)_2_D_3_ rescue of Caco-2 *ShPTEN* gland morphology (Figure 5D, 5E). Collectively, these findings show that mutationally-activated *KRAS* perturbs apical *PRKCZ*, impairs multicellular organization and suppresses 1,25(OH)_2_D_3_ rescue of Caco-2 Sh*PTEN* gland morphology.

### Translational and human studies

**Figure 6:**
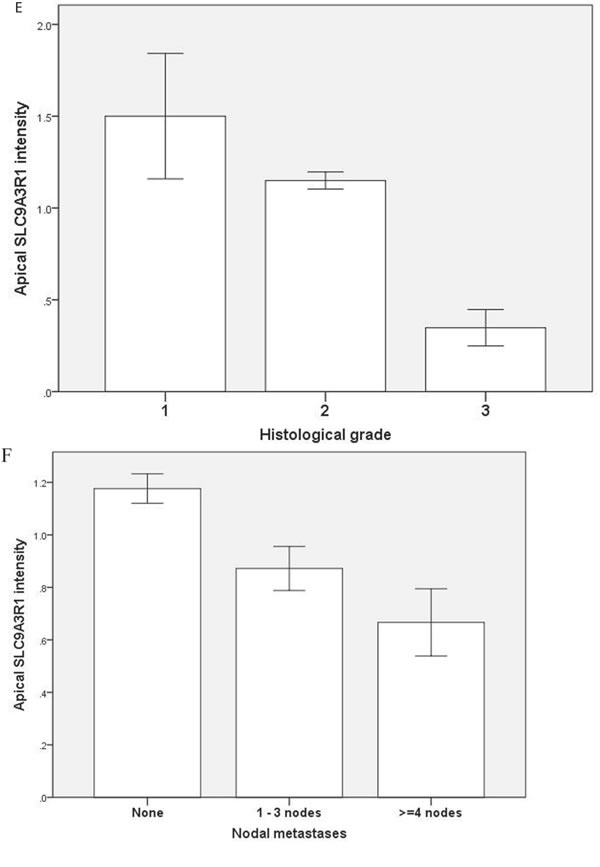
**A.** *PTEN-*deficient intestinal tumors show cribriform morphology. A H&E section of a small intestinal carcinoma driven purely by *PTEN-*deficiency, shows CM. Scale bar 100 μM. **B**. Relationship between log transformed ***PTEN*** RNA levels and IHC scores in 309 cores from 92 human CRCs (***r*** = 0.325; ***p*** < 0.01; Pearson's test). **C**. Apical *S****LC9A3R1*** and cribriform morphology. Apical *SLC9A3R1* intensity and CM in all CRC cores (open bars; *p* < 0.01) and in the wt *KRAS* CRC subset (shaded bars; *p* = 0.014 ANOVA). **D**. ***PTEN*** RNA and cribriform morphology. *PTEN* RNA levels (log values) and CM in all CRC cores (open bars; *p* = 0.056) and in the *KRAS* wt CRC subset (shaded bars; *p* < 0.01) ANOVA. **E**. Apical ***SLC9A3R1*** intensity and histological grade in all second cohort CRCs (***n*** = 92). Grade I - 1.50 ± 0.34; Grade II - 1.15 ± 0.05; Grade III - 0.35 ± 0.1;*p* < 0.01;ANOVA. **F**. Apical ***SLC9A3R1*** intensity and lymph node metastases in all second cohort CRCs (***n*** = 92). No nodal involvement - 1.18 ± 0.06; invasion of 1-3 nodes - 0.87 ± 0.08; invasion of ≥ 4 nodes - 0.67 ± 0.13; *p* < 0.01; ANOVA.

To investigate relationships between polarity signalling and tumor morphology, we conducted histologic, immunohistochemical or RNAscope *in situ* hybridization studies in murine or human intestinal tumors. We used an intestinal epithelial-specific *PTEN* knockdown murine model [34] to investigate morphology of intestinal cancers driven purely by *PTEN-*deficiency. Two small intestinal cancers developed after a long latency and showed cribriform morphology (Figure 6A). In whole sections of 35 human CRCs in cohort (i), CM was heterogeneously distributed and affected > 50% glandular structures in 11.3 ± 8.5/40 fields per tumor. CM involved < 20% CRC surface area and was detected at low power (LP) (x3) magnification in 19/35 CRC whole tumor sections (54%). In TMA studies of cohort (ii), CM was detected at LP microscopy in 131/306 CRC cores (43%) from 92 CRCs. CM was more frequent in grade I and II CRCs in both study cohorts (Supplementary Figure S6A). Thirty six CRCs were mutated at *KRAS* exons 12 or 13 while 56 had wt *KRAS*. *PTEN* mRNA and protein expression were assayed by RNAScope [35] and immunohistochemistry (IHC) respectively. *SLC9A3R1* IHC assays were also conducted as readout of apical *PRKCZ* activity [36]. We found that log-transformed *PTEN* RNAscope values (Supplementary Figure S6B*)*, *PTEN* IHC and *SLC9A3R1* IHC scores all correlated in human CRC (*PTEN* RNA *vs PTEN* IHC, r = 0.33; *p* < 0.01; *PTEN* RNA *vs SLC9A3R1* apical intensity r = 0.36; *p* < 0.01; *PTEN* IHC *vs SLC9A3R1* apical intensity r = 0.28; *p* < 0.01 Figure 6B). Apical *SLC9A3R1* intensity directly associated with CM in all CRCs (Figure 6C) but *PTEN* RNA expression associated with CM only in the *KRAS* wt subset (Figure 6D). Apical *SLC9A3R1* intensity had prognostic significance and inversely associated with histological grade (Figure 6E) and lymph node metastasis (Figure 6F).

Collectively, the above translational studies support the utility of 3D organotypic models for investigation of multiscale development of cancer morphology. As a manifestation of well- and moderately-differentiated CRC, CM associates with defective AP signalling and may represent an early or intermediate stage in a trajectory of cancer dedifferentiation (Supplementary Figure S6C).

## DISCUSSION

*PTEN* modulates the highly conserved apical *CDC42-PRKCZ-PARD* polarity complex [11, 12] that has a pivotal role in mitotic spindle orientation [7, 10, 29, 37], organization of epithelial architecture and tissue homeostasis [38]. *PTEN* regulates spindle orientation in nonpolarized cultured cells [13] and in this study we show *PTEN* regulation of spindle alignment in polarized Caco-2 cells during formation of simple colorectal glandular structures. Conversely, *PTEN-*deficiency induced spindle misorientation, epithelial stratification, apical membrane misalignment and formation of multiple abnormal lumens characteristic of cribriform morphology (CM). Furthermore, development of CM in Caco-2 Sh*PTEN* glands associated with increased gland cellularity and size, in accord with loss of *PTEN* antiproliferative activity [39]. SiRNA knockdown of *PTEN* also induced cribriform architecture in a different CRC cell type (SK-CO-15) that has the capacity for 3D organotypic growth [40]. Our findings thus provide a mechanistic template for *PTEN* regulation of mitotic spindle alignment, growth of simple or stratified epithelium, AM dynamics and organization of colorectal multicellular architecture.

Downstream of *PTEN*, the *CDC42-PRKCZ-PARD* apical complex [11] tightly orchestrates spindle dynamics [9] and cell polarization [10]. *PARD3* is essential for recruitment of *PRKCZ* to the apical surface, formation of the *PARD3-PRKCZ-PARD6* complex and for *CDC42* activation of *PRKCZ* [38]. These molecular interactions are implicated in multiple processes of epithelial organization [10]. Robust functional readouts of apical *PRKCZ* activity are provided by signal intensities of phospho-*PRKCZ* [9] or *SLC9A3R1* [36] at the apical domain. In this study, high apical p-*PRKCZ* or *SLC9A3R1* intensities in wt Caco-2 glands associated with appropriate subapical *PARD3* localization, correct spindle alignment and regular gland morphology. Conversely in *PTEN*-deficient Caco-2 Sh*PTEN* glands, we found reduced apical p-*PRKCZ* and *SLC9A3R1* intensities, *PARD3* mislocalization, spindle misalignment and aberrant multicellular glandular architecture. Hence, *PTEN* regulates components of the *CDC42/PRKCZ/PARD* apical polarity complex to control spindle orientation and 3D gland morphology.

Spindle microtubule regulatory kinases are controlled by nuclear *PTEN* [41] and nuclear import of *PTEN* is Ca^2+^-dependent [42]. Because 1,25(OH)_2_D_3_ may enhance *PTEN* expression [16] and promotes rapid Ca^2+^ signalling in a *VDR*-dependent manner [18], we tested effects of this secosteroid upon spindle regulatory machinery and gland morphology. 1,25(OH)_2_D_3_ upregulated *VDR* expression and enhanced *VDR* nuclear localization in Caco-2 and Caco-2 Sh*PTEN* cells, indicating Vit-D responsiveness of the model system. In Caco-2 Sh*PTEN* cells, 1,25(OH)_2_D_3_ treatment enhanced *PTEN* expression, increased *CDC42*-GTP levels, enhanced apical p-*PRKCZ* intensity and restored mitotic spindle orientation to levels comparable with wt Caco-2 cultures. 1,25(OH)_2_D_3_ treatment suppressed CM by restoring formation of epithelial monolayers and single lumens in Caco-2 Sh*PTEN* glands for the total sustainable interval of 3D culture growth (20 days). Conversely, cessation of 1,25(OH)_2_D_3_ treatment enabled rapid reappearance of CM. Vit-D has antiproliferative properties [43] and in this study, 1,25(OH)_2_D_3_ treatment also suppressed Caco-2 Sh*PTEN* gland cellularity. While previous studies of 1,25(OH)_2_D_3_ regulation of cribriform morphology are lacking, this secosteroid has been shown previously to suppress epithelial stratification in a human epidermis culture model [24] and promoted a symmetrical, circular shape of MCF10DCIS mammospheres [23].

The *PTEN/CDC42-PRKCZ-PARD* apical complex regulates a repertoire of morphogenic processes [10, 11, 29, 38]. We functionally dissected this signal transduction cascade to identify principal 1,25(OH)_2_D_3_-dependent effectors. While 1,25(OH)_2_D_3_ upregulated *PTEN*, it also rapidly activated *CDC42 by PTEN*-independent mechanisms and enhanced apical p-*PRKCZ* and *SLC9A3R1* signal intensities. Disruption of Caco-2 glandular morphogenesis by DN *CDC42* transfection or treatment with an allosteric *PRKCZ* pseudosubstrate inhibitor (*PRKCZ* I), as described previously [7, 29, 36], could not be reversed by 1,25(OH)_2_D_3_. Furthermore, restorative effects of 1,25(OH)_2_D_3_ on Caco-2 Sh*PTEN* gland morphology were blocked by *PRKCZ*I treatment. Collectively, these findings implicate *CDC42* and/or *PRKCZ* signalling in 1,25(OH)_2_D_3_ regulation of gland morphology.

Mitotic spindle machinery is governed in part by steroid hormone signalling [44]. 1,25(OH)_2_D_3_ is a multipotent secosteroid that regulates gene transcription and also induces rapid Ca^2+^ flux through L-type voltage-dependent Ca^2+^ channels (LTVDCCs) [31] and CaM-KII mediated release of Ca^2+^ from intracellular stores [32]. Ca^2+^ flux and CaM-KII signalling initiate juxtamembrane signal transduction [45], activate *CDC42* [19, 20] and modulate microtubule dynamics [21]. In this study, 1,25(OH)_2_D_3_ treatment enhanced intracellular Ca^2+^ concentration in Caco-2 cells that expressed LTVDCCs in basolateral membranes, as previously described [46]. 1,25(OH)_2_D_3_ treatment upregulated *CDC42* activity within minutes but took up to 24 hrs to enhance *PTEN* expression in Caco-2 cells. Furthermore, 1,25(OH)_2_D_3_ treatment upregulated *CDC42*-GTP in both *PTEN* ^+/+^ and *PTEN* ^−/−^ HCT116 colorectal cells. SiRNA *VDR* knockdown, blockade of LTVDCCs by NF treatment or inhibition of CaM-KII by KN-93 all suppressed 1,25(OH)_2_D_3_-mediated activation of *CDC42*. SiRNA knockdown of *VDR* or treatment with NF or KN-93 also blocked 1,25(OH)_2_D_3_-mediated activation of *CDC42* and reversal of CM in Caco-2 Sh*PTEN* glands. Taken together, these data implicate *VDR*, LTVDCC - and CaM-KII -mediated Ca^2+^ flux in Vit-D control of multicellular morphology, through *CDC42/PRKCZ* signalling.

Because mutant *KRAS* can impede Vit-D growth control [27, 47] and can modulate *VDR* signalling [29], we investigated its effects on 1,25(OH)_2_D_3_ pro-morphogenic activity. Transfection of *KRAS* wt Caco-2 cells [48, 49], with mutationally-activated *KRAS* V12 suppressed uniform localization of active *PRKCZ* at the apical membrane interface and induced formation of solid, cell-filled Caco-2 glands that lack a central lumen, as previously reported [33]. Furthermore, *KRAS* V12 antagonized 1,25(OH)_2_D_3_ rescue of Caco-2 Sh*PTEN* gland morphology. Hence, mutationally-activated *KRAS* impedes *PRKCZ* apical localization, disrupts CRC multicellular architecture and inhibits Vit-D promorphogenic activity, in 3D models.

To explore the translational relevance of our findings, we investigated tumor formation in an intestinal-epithelial specific *PTEN*-deficient murine model [34] and assessed polarity signalling against CM in 2 human CRC cohorts. In the murine model, small intestinal cancers driven purely by *PTEN*-deficiency [34] developed CM. These findings accord with previous reports of CM in various cancers of *PTEN*-haploinsufficient mice [50]. In human studies of cohort (i), we found CM on low power microscopy in 54% CRCs and heterogenous CM distribution whole tumor sections. Because *PTEN* deficiency and mutationally-activated *KRAS* can synergistically co-regulate tumor morphology in transgenic mice [51], we studied a larger series of 92 *KRAS* genotyped CRCs (cohort ii) and found CM on low power microscopy in 43% CRCs. CM associated with grade I and II CRCs in both cohorts, consistent with an early transition state during a trajectory of CRC dedifferentiation. In accord with previous findings [52], we found no relationship between *PTEN* expression and *KRAS* mutational status in human CRC. However, *PTEN RNA* directly associated with CM in *KRAS* wt tumors but not in the *KRAS* mutant CRC subgroup, nor in the total CRC series of cohort (ii). These findings suggest that *PTEN-KRAS* epistatic interactions may influence human CRC morphology.

Downstream of *PTEN*, apical *PRKCZ* represents a central morphogenic effector within the apical polarity complex [11, 36]. Apical *SLC9A3R1* intensity provides a robust readout of apical *PRKCZ* activity in 3D models [36] and can be reliably assessed in human formalin-fixed paraffin embedded (FFPE) colorectal specimens [36, 53]. In cohort (ii) human CRCs, we found positive correlations between *PTEN* RNAscope, *PTEN* IHC and apical *SLC9A3R1* IHC intensity. We and others have shown that apical *SLC9A3R1* intensity [36, 53] and *PTEN* expression [54, 55] are substantively higher in normal colonic mucosa than in CRC [36, 53-55]. In CRCs of the present study, we found higher expression of apical *SLC9A3R1* intensity in cribriform as opposed to non-cribriform CRCs, consistent with CM as an early or intermediate stage in a trajectory of cancer dedifferentiation. A similar rationale may explain the higher *PTEN* RNA expression in cribriform CRC than in non-cribriform *KRAS* wt CRCs.

Compelling experimental, epidemiological and clinical data show that Vit-D controls inception and progression of CRC [25, 26]. However, there is a fundamental gap between discovery of Vit-D anticancer activity and identification of mechanistic biomarkers needed to exploit its full clinical potential. Strikingly, our findings show that 1,25(OH)_2_D_3_ controls subcellular, cellular and multicellular scales of tissue assembly to suppress CM. Hence, 1,25(OH)_2_D_3_ anticancer effects may be mediated in part by Ca^2+^/CaM-KII-dependent reprogramming of polarization machinery to suppress oncogenic disruption of homeostatic multicellular architecture. Our study shows that *KRAS* mutation indicates Vit-D-resistance. Conversely, apical *SLC9A3R1* intensity provides readout of *PRKCZ* [36] a key morphogenic effector of the *PTEN/CDC42/PRKCZ* pathway [11], has prognostic relevance in human CRC and predicts 1,25(OH)_2_D_3_ control of gland morphology. Apical *SLC9A3R1* intensity is suppressed by mutationally-activated *KRAS* in 3D models and associates with CM in both *KRAS* mutant and wt human CRCs.

Globally, cancer affects over 12 million new patients each year [56]. Cancer morphology has been a gold-standard for diagnosis and outcome prediction since the time of Virchow [57] but has remained a mechanistic “black box” with few advances and almost no literature exploring its pathobiology. Our MS now untangles the molecular framework of cribriform morphology in 3D CRC models, shows Vit-D suppression of CM evolution *via* core polarization machinery and conducts translational and clinical studies that support model predictions. We also identify biomarkers of Vit-D resistance (*KRAS* mutation) and promorphogenic effects (apical *SLC9A3R1*) for use in future clinical trials.

## MATERIALS AND METHODS

### Reagents and antibodies

Laboratory chemicals were purchased from Sigma-Aldrich, Dorset, England unless otherwise stated. Antibodies included mouse anti-*PTEN* (Cell Signaling, Danvers, MA, USA and Dako anti-*PTEN* clone 6H2.1), mouse anti-*SLC9A3R1*, Lifespan Biosciences, Seattle, WA, USA), anti-*CDC42* and anti-*CTNNB1* (also known as β-catenin - Cell Signaling, Danvers, MA, USA), anti-CaV1.3 antibody against the α subunit of LTVDCCs [58], anti-*GAPDH* (glyceraldehyde-3-phosphate dehydrogenase (ab8245); anti-*VDR* (ab54373) and rabbit anti-phospho-*PRKCZ* [Thr 560] (all from Abcam Cambridge, MA, USA). For confocal microscopy, primary antibodies were used in conjunction with Alexa Fluor 568 (anti-rabbit) and Alexa Fluor 488 (anti-mouse; Molecular probes, Invitrogen, Carslbas, CA, USA).

### Cell lines

Stable *PTEN*-deficient Caco-2 Sh*PTEN* cells were generated by transfection of parental Caco-2 cells with replication-defective retroviral vectors encoding *PTEN* short hairpin RNA (shRNA), using the Phoenix™ retroviral expression system (Orbigen, San Diego, CA USA), as previously described [12, 36]. Transient SiRNA *PTEN* knockdown was conducted in SK-CO-15 colorectal cells (gift from Dr F Real, Madrid) that have the capacity for 3D organotypic growth [40]. *PTEN* ^+/+^ and *PTEN* ^−/−^ HCT116 cells were used in signalling assays, as previously described [59]. Caco-2 clones and SK-CO-15 cells were propagated in two-dimensional (2D) cell culture flasks in MEM (modified Eagle's medium) supplemented with 10% FCS, 1mM non- essential amino acids and 1mM L-glutamine at 37°C in 5% CO_2_. *PTEN* ^+/+^ and *PTEN* ^−/−^ HCT116 cells were cultured in McCoys 5A media supplemented with 10% FCS, 1mM L-glutamine and 1mM sodium pyruvate, as previously described [12, 36].

### Three-dimensional (3D) cultures

Development of multicellular architecture was assessed in *PTEN*-expressing Caco-2, *PTEN*-deficient Caco-2 Sh*PTEN* cells, parental *PTEN*-expressing SK-CO-15 cells and a subclone rendered *PTEN*-deficient by SiRNA knockdown, in organotypic cultures. Cells were cultured and embedded in Matrigel matrix (BD Biosciences, Oxford, UK), then imaged by confocal microscopy during progressive development of multicellular glandular architecture, as previously described [12, 36]. SK-CO-15 cells express apical membrane markers at low level [60] and apical *SLC9A3R1*, *PRKCZ* or *p-PRKCZ* were undetected in these cells, in this study. Cribriform morphology (CM) was defined as multiple aberrant lumens surrounded by abnormal stratified epithelium in 3D multicellular structures in culture and in tumors [6]. Effects of transfections or treatments on glandular morphology of 3D cultures were assessed against endpoints of CM or individual features of epithelial configuration (columnar or stratified) or single central lumen formation.

### Transfections and treatments

Caco-2 and/or Caco-2Sh*PTEN* 3D cultures were transfected with mutant *CDC42* constructs, as previously described [12, 36] and/or treated by 1,25(OH)_2_ D_3_ (10^−7^M), inhibitors of L-type calcium channels (nifedipine) [61], calcium calmodulin-dependent protein kinase II (CaMKII) [KN-93] [62] or a myristoylated *PRKCZ* pseudosubstrate peptide containing a membrane-targeting myristoylation tag that functions as an effective *PRKCZ* pseudosubstrate inhibitor (*PRKCZ*I) [63].

### Intestinal-epithelial specific *PTEN*-deficient murine model

All animal procedures were conducted in accordance with local and national regulations. Mice were generated, housed, and genotyped, and Cre activity was induced as previously described [34]. A total of 30 Ah::CreERT^T+^/^0^;Pten^F/F^ mice and 29 Ah::CreERT^T+/0^;Pten^+/+^ mice were enrolled into cohorts for prolonged follow up. Tissues were harvested, fixed, and processed according to standard protocols, as previously described. [34]. Animals were monitored closely for symptoms of disease, and were then necropsied as previously described [34]. The morphology of tumors arising in *PTEN*-deficient murine intestinal epithelium was assessed by H&E histology. Cribriform morphology was assayed as previously defined [6].

### Human colorectal cancer studies

We conducted 2 separate studies of polarity signalling against cribriform morphology (CM) in human colorectal cancer (CRC). We used anonymised formalin fixed, paraffin embedded (FFPE) samples from (i) 35 patients with non-genotyped CRCs and (ii) 92 patients with *KRAS*-genotyped CRCs. We assessed CM at low power microscopy according to previously defined criteria [6] in both study cohorts. To assess CM heterogeneity, we scored CM in 40 fields per tumor at 20x magnification across whole tumor sections in cohort (i). Scores of 0, 1 and 2 were given for CM involvement of < 10%, 11-50% and > 50% CRC epithelium per field. In cohort (ii), specimens were arranged in tissue microarrays (TMAs). To assess polarity signalling in CRC FFPE specimens, we assessed *PTEN* RNA expression by RNAscope *in situ* hybridization [35]. We assessed apical *SLC9A3R1* intensity by immunohistochemistry (IHC) as readout of apical *PRKCZ* activity as outlined previously [36], in FFPE specimens of both study cohorts. To assess *PTEN* protein expression, *PTEN* IHC was also assessed in cohort (ii) TMAs. Samples used in this research were released from the Northern Ireland Biobank (NIB13-0090), approved by the Office of Research Ethics Committees Northern Ireland (Reference number 11/NI/0013/-/NIB13-0090).

### Data analysis

Descriptive statistics were expressed as the mean ± sem. Statistical analyses were by one or two-way ANOVA or Student's t test using SPSS for Windows release 22.0 (IBM Corp, NY, USA) or Graphpad Prism software (v4.02; Graphpad CA 92037 USA). Scatterplots and bar charts were used for display of quantitative numerical or categorical data. *PTEN* RNA values were log transformed to provide a normal distribution.

## SUPPLEMENTARY MATERIALS FIGURES


