# Supplementary material for: Vitamin D3 suppresses morphological evolution of the cribriform cancerous phenotype
Source: Oncotarget. 2016 Apr 20;7(31):49042–64. doi: 10.18632/oncotarget.8863 (PMC5226489; doi:10.18632/oncotarget.8863)
Supplement: Supplementary file 1 [file oncotarget-07-49042-s001.pdf]

Vitamin D_3 suppresses morphological evolution of the cribriform cancerous phenotype

Supplementary Material

Intracellular Ca^{2+}-fluorescence imaging Caco-2 cells were loaded with 2 µM fluo-4AM (Molecular Probes, Life Technologies), washed in indicator-free medium and imaged with a Nikon 80i upright epifluorescent microscope (Nikon UK Ltd., Surrey, UK) using a x60W Fluor NA 1.0 objective lens. Fluorescence was imaged at 5 fps (2x2 binning) with an EMCCD-imaging system (DQC-FS, Nikon) and recorded to a personal computer running WinFluor software (v3.2.25, University of Strathclyde). The background-corrected fluorescence (F) at any time point was normalised to baseline fluorescence (F_0) which was average fluorescence in the cell of interest, during the first 100 frames of recording. Amplitude ($\Delta F/F_0$) of $1,25(OH)_2D_3$ - evoked increase in fluorescence was measured in WinFluor and Clampfit (pClamp, v10.3) and analysed in Microsoft Excel and Prism software (v4.02, Graphpad).

***GST*-PAK (Glutathione sulfotransferase - p21 activated kinase) pulldown assays, protein extraction and Western blotting** These procedures were conducted as previously reported [1, 2].

Small interfering RNA (SiRNA) knockdown of *PTEN* RNA interference-mediated knockdown of PTEN was performed by transfection of synthetic duplex RNA oligonucleotides, using Oligofectamine (Invitrogen, Carlsbad, CA, USA) according to manufacturers' instructions, as we have previously described [1, 2]. Briefly, On-Target plus SMART pool *PTEN* (catalog no. L-003023-00, Dharmacon, Fisher Scientific, Dublin, UK) siRNA oligonucleotides were used. On-Target plus non-

targeting siRNAs were used as controls (catalog no. D-001810-01-05, Dharmacon). Oligofectamine and Opti-Mem serum-free medium) [Invitrogen] were mixed in 1 : 3 ratio, incubated at room temperature for 5 min and then added to siRNA, which was prediluted in Opti-Mem to final concentration of 50 nM and then added to cultures. Cells were grown in 90 mm dishes and were transfected at 50–60% confluence by incubation in the above mixture. The medium was changed after 6 h and cells were harvested after 48 h. *PTEN* expression was assayed by western blot.

Imaging of gland morphology in 3D cultures Briefly, glands were permeabilised with 0.5%Triton x 100, rinsed and blocked in buffer containing 0.1% bovine serum albumin and 5% goat serum. Glands were incubated overnight in primary antibodies. Apical *p-PRKCZ* (Thr 560) or *SLC9A3R1* immunofluorescence intensities were each used as readouts of apical *PRKCZ* activity [2, 3]. DNA was stained by DAPI and chamber slides were mounted using Vectashield medium (Vector Scientific, Belfast, NI, UK). Sequential 3-color scan images were taken at gland midsections at room temperature using a Leica SP5 confocal microscope on a HCX PL APO lambda blue 63x 1.40 oil immersion objective at 1x zoom. Images were collected, processed, merged and scale bars added using LAS AF Leica Confocal Imaging Software (Leica Biosystems, Milton Keynes, UK). The ImageJ toolkit (NIH) was used to measure apical signal intensity of p-*PRKCZ* or S*LC9A3R1* in untreated and treated glands. Total apical domains surrounding gland lumens were captured in rectangular ImageJ selection windows and fluorescence quantified, plotted and statistically analysed. X/y or x/z focal plane images were generated from Z-Stack images with ImageJ software, as previously described [3]. In assessment of cell number, individual cells were identified by DAPI positivity and counted in at least 50 glands for each

condition, in triplicate. Gland diameters were measured using ImageJ and surface areas calculated.

Spindle orientation assays For these studies, Caco-2 and Caco-2 Sh*PTEN* glands were cultured in Matrigel for 4 days, fixed with 4% PFA (paraformaldehyde) and stained with α-tubulin and p-*PRKCZ* (T-560) primary antibodies. Gland midsections were imaged by confocal microscopy to identify cells containing well-formed mitotic spindles, during metaphase or anaphase. Lines connecting each spindle extremity were drawn using ImageJ and the line centre was considered as the spindle midpoint. Angles between spindle axes and lines connecting spindle midpoints to gland centres were measured, as outlined previously [3].

Assessment of cell stratification Caco-2 and Caco-2 Sh*PTEN* glands were cultured in Matrigel for 4-12 days and mid-sections were imaged by confocal microscopy. Cell stratification was recognized by at least 2 layers of epithelial cells with nuclear-nuclear planes orientated parallel to cells long axes and could be focal, circumferential or arranged around multiple abnormal gland lumens. At least 50 glands were assessed for each condition per experiment and were blinded to treatment.

Patient samples Formalin-fixed, paraffin-embedded (FFPE) colorectal primary tumours were collected from 35 non-consecutive patients to provide a range of tumour differentiation by conventional histological grading criteria [4]. All histomorphic data from haematoxylin and eosin (H&E) and *SLC9A3R1*-stained slides were reviewed and clinical data were obtained from corresponding reports, as

previously outlined [2]. Cribriform morphology was assessed in 40 fields per H&E stained tumour section. To investigate *KRAS* mutational status, we collected a further 92 FFPE primary CRC samples that had been *KRAS* genotyped (see below). Clinicopathologic information including histological grading, Duke's stage, depth of cancer invasion and lymph node metastases were recorded. All identifiable details from clinical and histopathological information were anonymized and the study was approved by the Belfast HSC Trust Research Office and the Regional Ethics Committee for Northern Ireland.

***KRAS* mutational analysis** DNA was extracted from 92 CRCs using the DNeasy Blood & Tissue kit (Qiagen, UK) according to the manufacturer's instructions. Primers for amplification and pyrosequencing were designed using Pyrosequencing Assay Design Software (Biotage AB, Uppsala, Sweden). KRAS codons 12 to 13 were amplified in one polymerase chain reaction (PCR). PCR products were pyrosequenced on a PyroMark Q24 MDx system (Qiagen) according to the manufacturer's protocol. All mutations were verified by Sanger sequencing, as previously described [5].

Tissue Microarray (TMA) FFPE specimens from 92 *KRAS* - genotyped CRCs were subsequently sampled to construct 2 TMA blocks. Each whole CRC section block was assessed histologically and 2-4 x 1.0 mm cores were taken from each. Where possible, 2 cores were selected to contain cribriform morphology (CM) and 2 without CM, in each tumour. In CRCs lacking CM, only non-CM cores were taken. Cores from each tumour were subsequently placed horizontally adjacent within the same TMA block. The two TMA blocks contained 309 cores and were used to generate

5µM tissue sections. Sections were stained for H&E and by IHC for *PTEN* using two antibodies (DAKO mouse monoclonal Clone 6H2.1 and Cell signalling 138G6; rabbit monoclonal). *SLC9A3R1* IHC was carried out as previously described [2]. *PTEN* in situ hybridization was conducted by RNAscope using Ubiquitin C (*UBC*) RNA as a positive control for RNA integrity and a bacterial DapB probe as negative control (*vide infra*).

Assessment of CRC morphology Haematoxylin and eosin and *SLC9A3R1* IHC stained whole FFPE sections from 35 CRCs and TMAs from 92 *KRAS* genotyped CRCs were scanned at 40X magnification into an APERIO ScanScope CSO digital slide scanner (Leica Biosystems, Milton Keynes, UK) with BRIGHTFIELD setting. The resulting SVS image files were uploaded into a PathXL Digital Pathology account (PathXL LTD, Belfast, NI). Tumour morphology was assessed in digital images by at least 2 observers (JMcC, RH, FCC and/or MBL) in whole mount sections and in TMA cores. Tumours were defined as cribriform if the characteristic morphology [6] was detected at low power (x3) histology. The extent of cribriform morphology was assessed in whole mount sections of 35 CRCs in up to 40 fields at 10X magnification across the tumour surface area. Scores were assigned for cribriform change in <10% to >50% glandular structures in each field.

***PTEN* RNAscope assays** - The RNAscope probe targeting *PTEN* mRNA was obtained from Advanced Cell Diagnostics and detection of *PTEN* mRNA expression was performed using the RNAscope 2.0 High- Definition (HD)-BROWN assay in accordance with manufacturer’s instructions (Advanced Cell Diagnostics [ACD], CA 94545, USA). Chromogenic RNAScope was performed on FFPE human CRC

wholemount sections of 35 CRCs and TMAs from 92 *KRAS* genotyped CRCs using company protocols. Briefly, sections were cut at 4µm, air dried overnight, baked at 60^0C for 1hr, dewaxed and air-dried before pre-treatments. For all probes the blocks were subjected to a standard pre-treatment protocol. Three RNAScope probes from ACD were used in this study, namely Hs-*PTEN* (310121 Accession # NM_000314, nucleotides 2698-3283), positive control probe HS-*UBC* (310041 Accession # NM_021009) and negative control probe bacterial *DapB* (310043 Accession # EF191515). Detection of specific probe binding sites was with RNAScope 2.0 HD Reagent kit - brown from ACD (Cat. No. 310035). For semi-quantitative microscopical evaluations of *PTEN* mRNA detection by RNAScope a 3-tier scoring system was developed: + = few spots in most cells; ++ = moderate number of spots in all cells; +++ = high number of spots in all cells. Image analysis of selected regions of interest within *PTEN*-probe labelled tumours was performed using Spotstudio™ Software from Advanced Cell Diagnostics Hayward, CA 94545 USA, with user-defined thresholds after slides were scanned using Aperio scanner (Leica Biosystems, Milton Keynes, UK) at x40 resolution. Results for these selected cases were expressed as average number of spots per cell. *PTEN* RNA expression was log transformed to provide a normal distribution and assessed against cribriform morphology and other pathological variables. *PTEN RNA* expression was also compared in cribriform and non-cribriform regions of interest areas within individual tumours and between tumours.

***PTEN* immunohistochemistry** Immunohistochemistry was performed on 4 *µ*m sections of formalin-fixed, paraffin-embedded tumors. Following deparaffinization and rehydration of the tissues sections, antigen retrieval was performed at 100° C for

20 min with Tris-EDTA buffer, pH 6.0 or using microwave treatment in 10 mM citrate buffer. Endogenous peroxidase was blocked with 3% peroxide for 5 min. We tested two primary *PTEN* antibodies (Cell Signaling, Danvers, MA, USA and Dako anti-*PTEN* clone 6H2.1). The anti-*PTEN abs* were applied at 1:100 dilution. Primary antibody detection was carried out using a polymer system (Bond Polymer Refine Detection, Leica). Staining development was achieved by incubation with DAB and DAB Enhancer (Leica). Because IHC results appeared comparable with either *PTEN* ab, semi-quantitative assessment of *PTEN* expression was scored by skilled observers (JMcC and MBL) in sections stained by the Cell Signaling ab only. Staining intensity scores of 0,1 and 2 for absent, moderate and strong *PTEN* expression were applied. Final IHC scores were derived from the product of staining intensity (0-2) and % tumour occupied by epithelial cells with a specific intensity.

***SLC9A3R1* immunohistochemistry** Assays were conducted as previously described [2]. Apical localization of *SLC9A3R1* was assessed in CRC sections, scored 0-2 and final scores derived as outlined above for total *PTEN* expression.

References to Supplementary Materials and Methods

1. Jagan I, Fatehullah A, Deevi RK, Bingham V and Campbell FC. Rescue of glandular dysmorphogenesis in PTEN-deficient colorectal cancer epithelium by PPARgamma-targeted therapy. Oncogene. 2013; 32(10):1305-1315.
2. Jagan IC, Deevi RK, Fatehullah A, Topley R, Eves J, Stevenson M, Loughrey M, Arthur K and Campbell FC. PTEN phosphatase-independent maintenance of glandular morphology in a predictive colorectal cancer model system. Neoplasia. 2013; 15(11):1218-1230.
3. Jaffe AB, Kaji N, Durgan J and Hall A. Cdc42 controls spindle orientation to position the apical surface during epithelial morphogenesis. J Cell Biol. 2008; 183(4):625-633.
4. Jass JR, Atkin WS, Cuzick J, Bussey HJ, Morson BC, Northover JM and Todd IP. The grading of rectal cancer: historical perspectives and a multivariate analysis of 447 cases. Histopathology. 1986; 10(5):437-459.

5. Van Schaeybroeck S, Kalimutho M, Dunne PD, Carson R, Allen W, Jithesh PV, Redmond KL, Sasazuki T, Shirasawa S, Blayney J, Michieli P, Fenning C, Lenz HJ, Lawler M, Longley DB and Johnston PG. ADAM17-dependent c-MET-STAT3 signaling mediates resistance to MEK inhibitors in KRAS mutant colorectal cancer. Cell Rep. 2014; 7(6):1940-1955.
6. Fletcher C. (2013). Chapter 9 Tumors of the Small and Large Intestines, Including Anal Canal Diagnostic histopathology; Fourth Edition
(Boston: Elsevier).

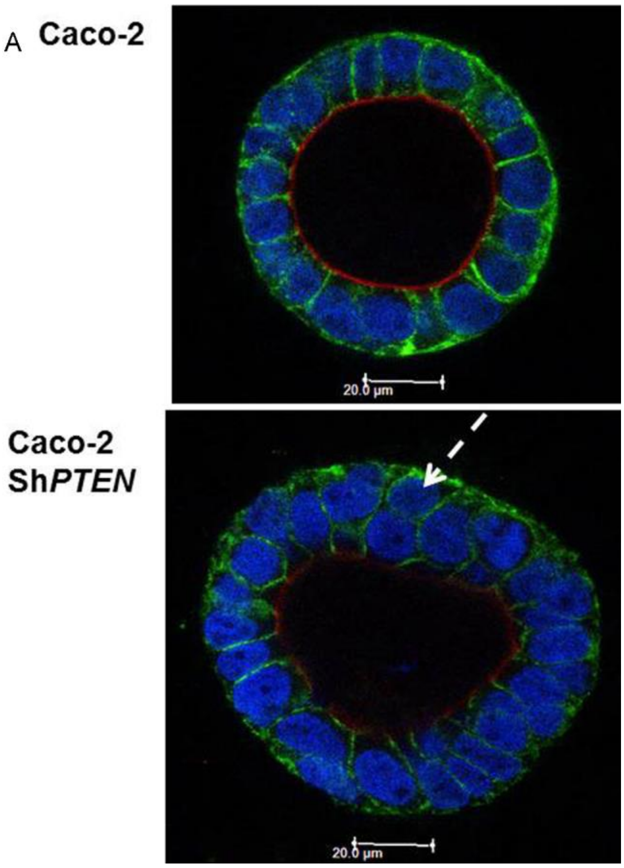
A
Caco-2
20.0 µm
Caco-2
ShPTEN
20.0 µm

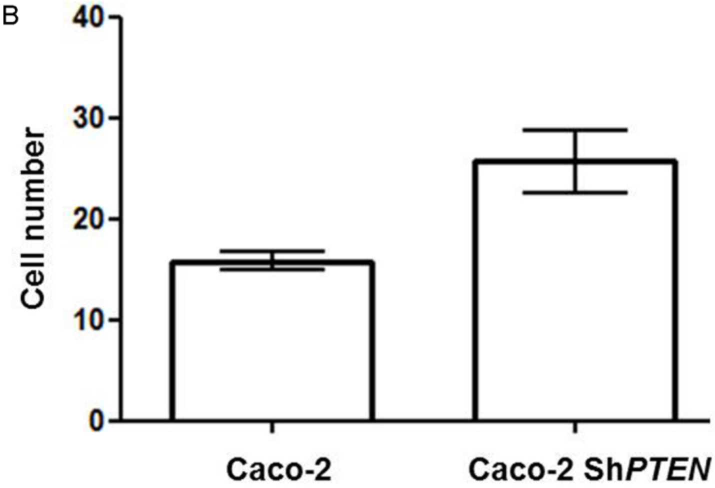
B
Cell number
40
30
20
10
0
Caco-2
Caco-2 ShPTEN

C

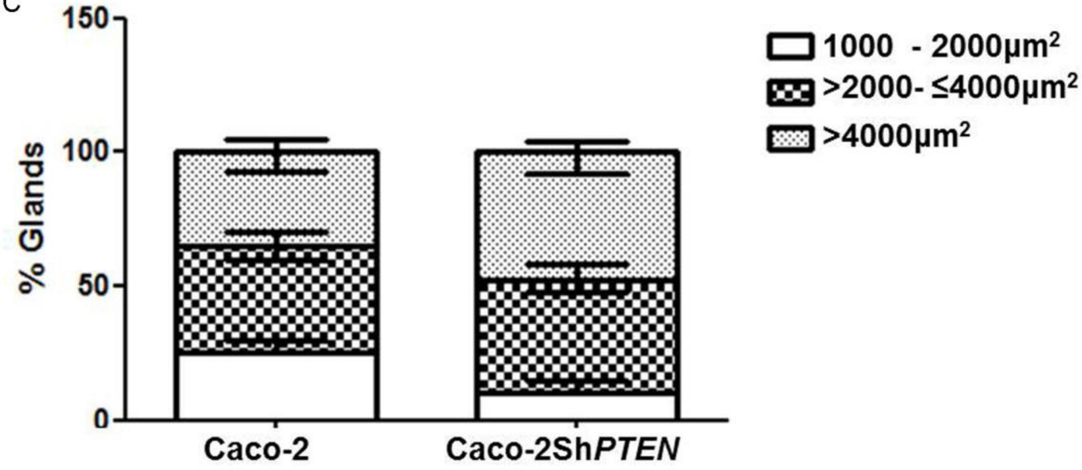
150
100
50
0
% Glands
1000 - 2000µm2
>2000- ≤4000µm2
>4000µm2
Caco-2
Caco-2ShPTEN

D

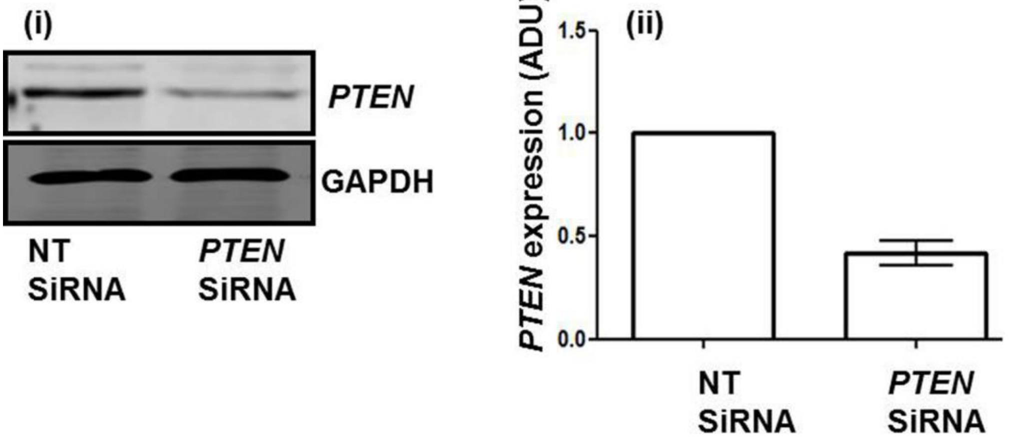
(i)
PTEN
GAPDH
NT SiRNA
PTEN SiRNA
(ii)
1.5
1.0
0.5
0.0
PTEN expression (ADU)
NT SiRNA
PTEN SiRNA

E

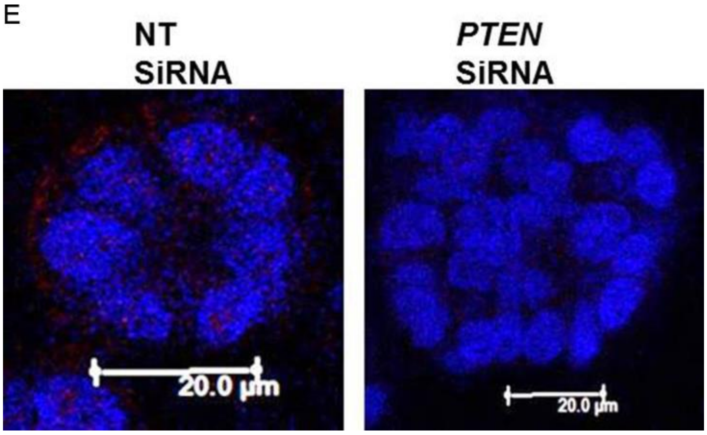
NT SiRNA
PTEN SiRNA
20.0 µm
20.0 µm

F

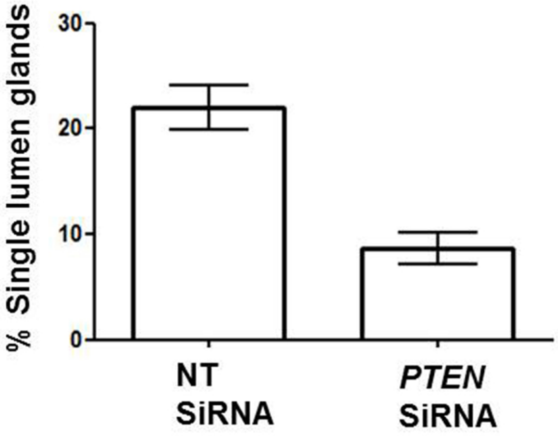

G

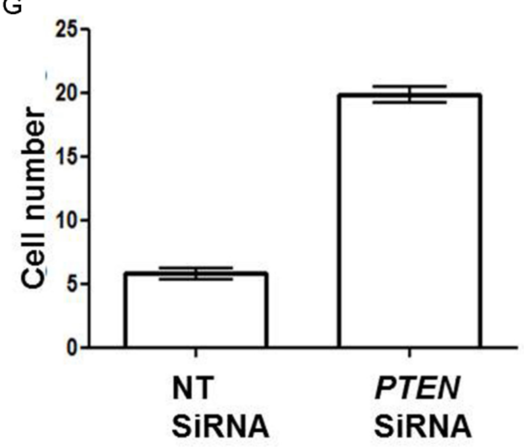

Supplementary Fig 1. A Effects of *PTEN* knockdown on focal epithelial stratification. An epithelial monolayer and stratified epithelium are shown in Caco-2 and Caco-2 Sh*PTEN* glands respectively at 12 days. In the Caco-2 Sh*PTEN* gland shown, focal stratification (broad interrupted white arrow) was found in late stage glands associated with a single lumen. Scale bar - 20 μM. **B** Summary effects of *PTEN* knockdown on gland cellularity. Values shown for Caco-2 and Caco-2 Sh*PTEN* glands at 12 days of culture (15.8 ± 0.85 *vs* 25.6 ± 3.05 cells per gland; p<0.01;Students t test). **C** Summary effects of *PTEN* knockdown on gland size. Values shown represent percentages of Caco-2 *vs* Caco-2Sh*PTEN* glands with surface areas (calculated by πr^2), between 1000-2000, >2000 - ≤4000 and >4000

μm^2 at 12 days of culture and were 25.0 ± 5.0 *vs* 10 ± 5; 40.0 ± 5.0 *vs* 42.0 ± 6.0 and 35.0 ± 5.0 vs 48.0 ± 4.0 respectively; <0.01;ANOVA). **D** SiRNA knockdown of *PTEN* in SK-CO-15 cells shown in immunoblot (i) and represented graphically (ii). Values (0.42 ± 0.06) are expressed as fold change relative to NT SiRNA transfection.**E** SiRNA knockdown of *PTEN* induced CM identified by multiple lumens and stratified epithelium, in 3D SK-CO-15 cultures. Apical *SLC9A3R1, PRKCZ* and p-*PRKCZ* were undetected by confocal immunofluorescence in SK-CO-15 cells. **F** Summary effects of *PTEN* knockdown on single lumen formation in SK-CO-15 glandular structures (glands) at 4 days of culture. **G** Summary effects of *PTEN* knockdown on SK-CO-15 gland cellularity at 4 days of culture (NTSiRNA = 5.8 ± 0.44; *PTEN* SiRNA = 20.0 ± 0.63; p<0.01; Student's t test).

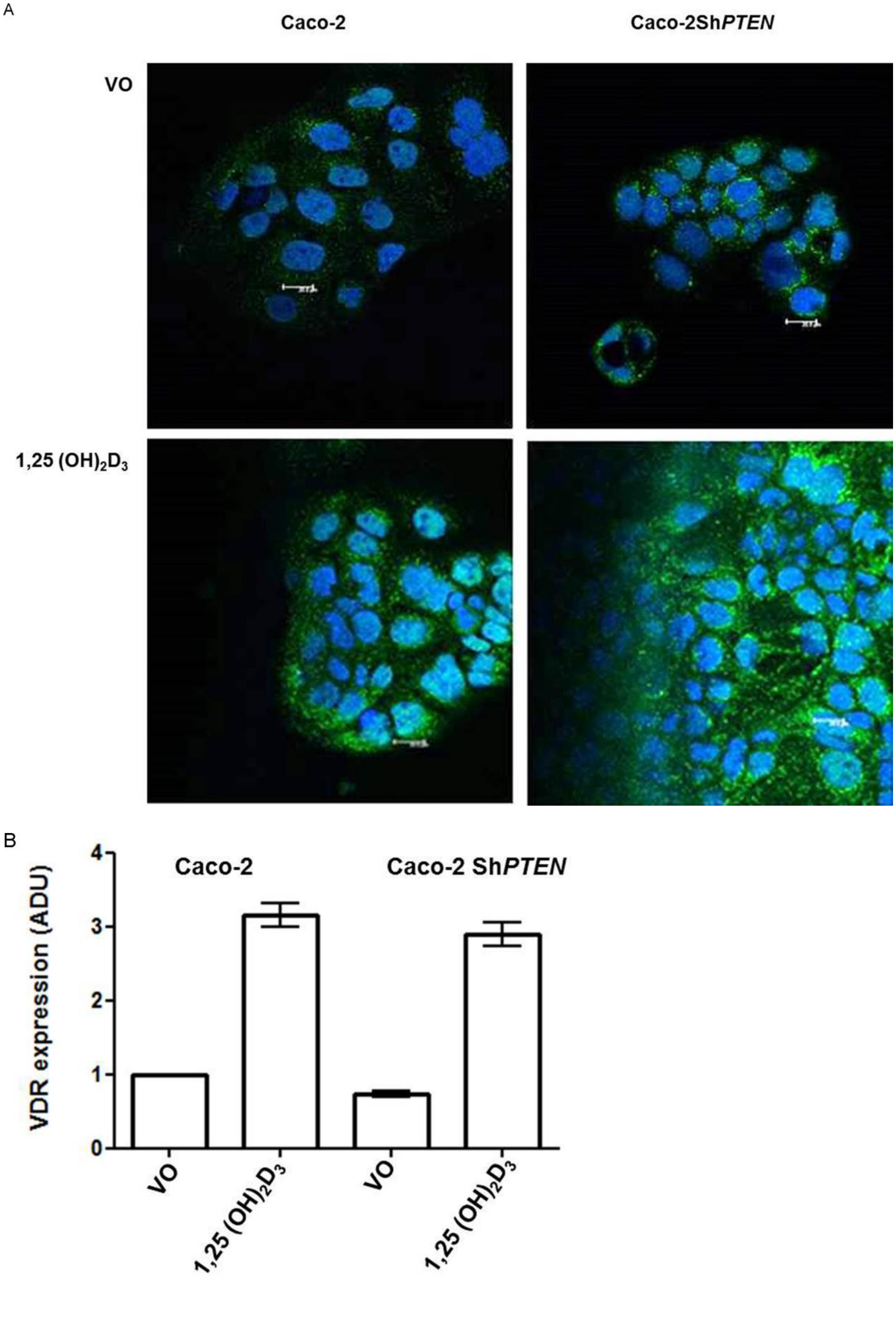

A
Caco-2
Caco-2ShPTEN
VO
1,25 (OH)2D3
B
Caco-2
Caco-2 ShPTEN
VDR expression (ADU)
0
1
2
3
4
VO
1,25 (OH)2D3
VO
1,25 (OH)2D3

C

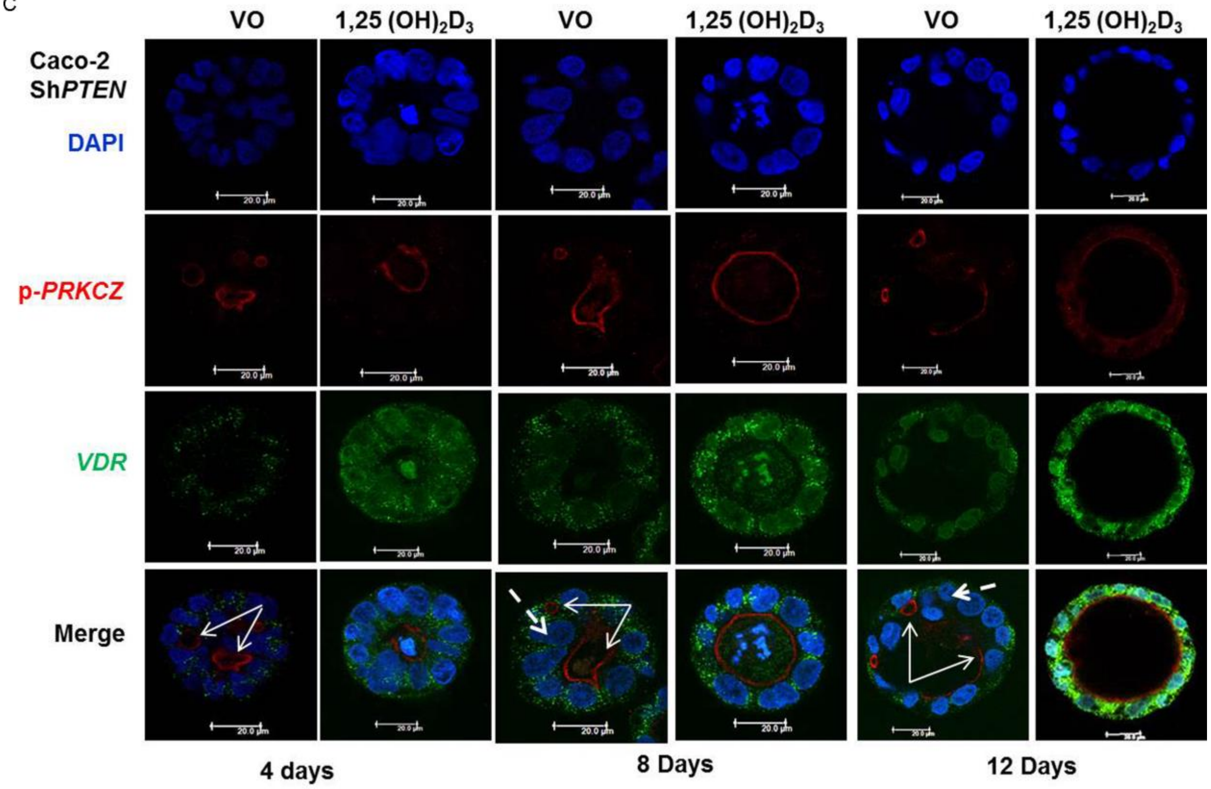
VO
1,25 (OH)2D3
VO
1,25 (OH)2D3
VO
1,25 (OH)2D3
Caco-2
ShPTEN
DAPI
p-PRKCZ
VDR
Merge
4 days
8 Days
12 Days

D

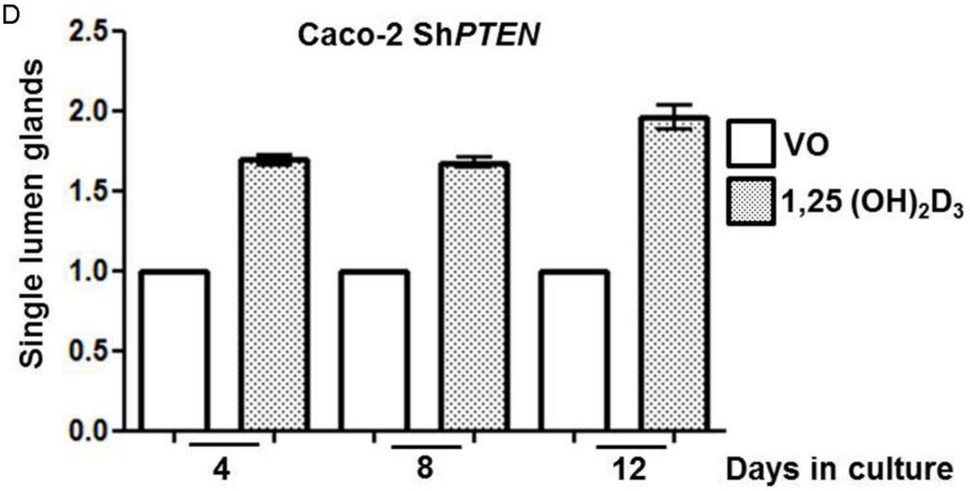
Caco-2 ShPTEN
Single lumen glands
2.5
2.0
1.5
1.0
0.5
0.0
VO
1,25 (OH)2D3
4
8
12
Days in culture

E

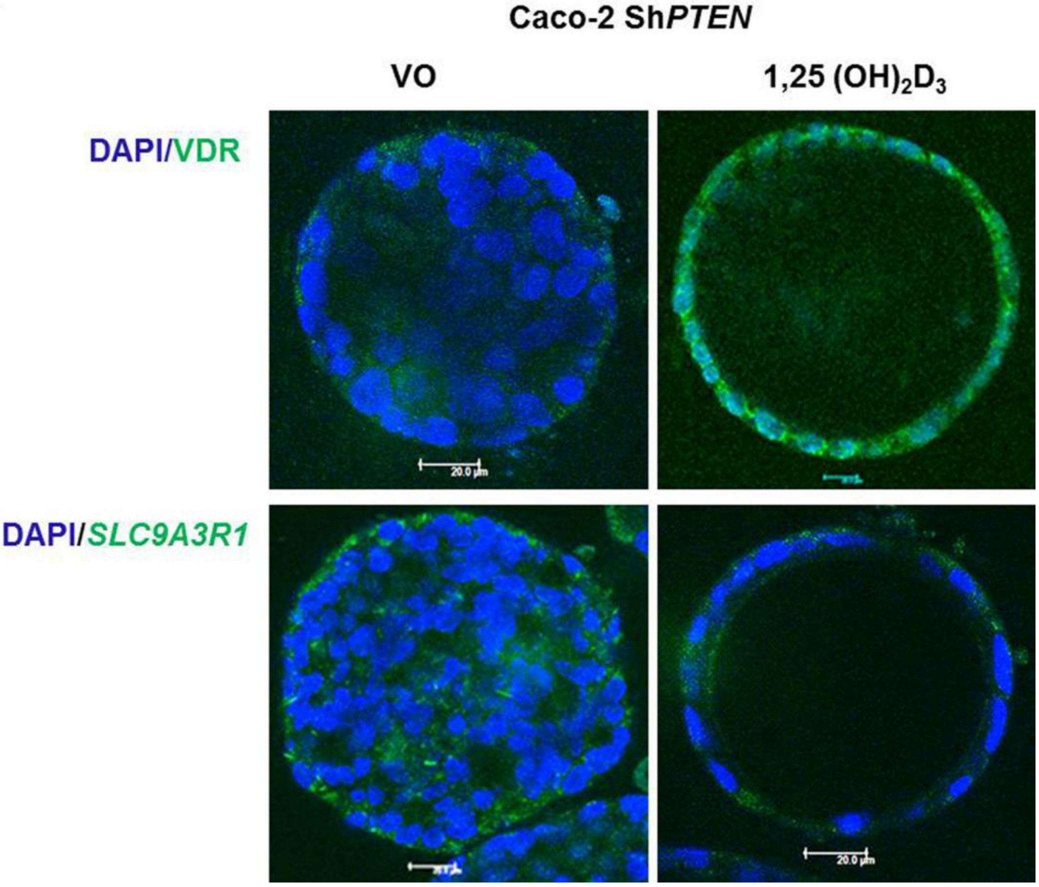
Caco-2 Sh*PTEN*
VO
1,25 $(OH)_2D_3$
DAPI/VDR
DAPI/*SLC9A3R1*
20.0 µm
20.0 µm

F

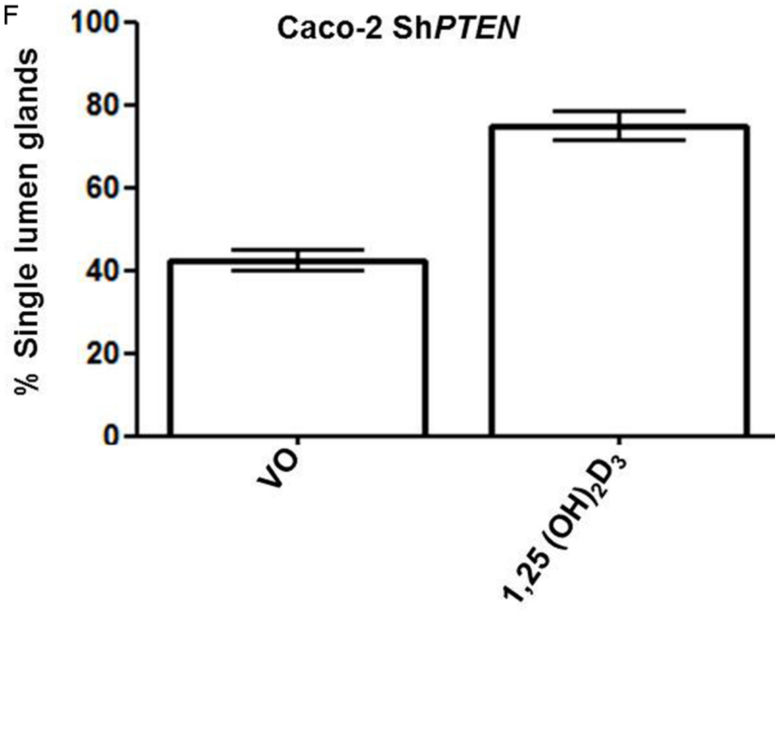
Caco-2 Sh*PTEN*
% Single lumen glands
100
80
60
40
20
0
VO
1,25 $(OH)_2D_3$

G

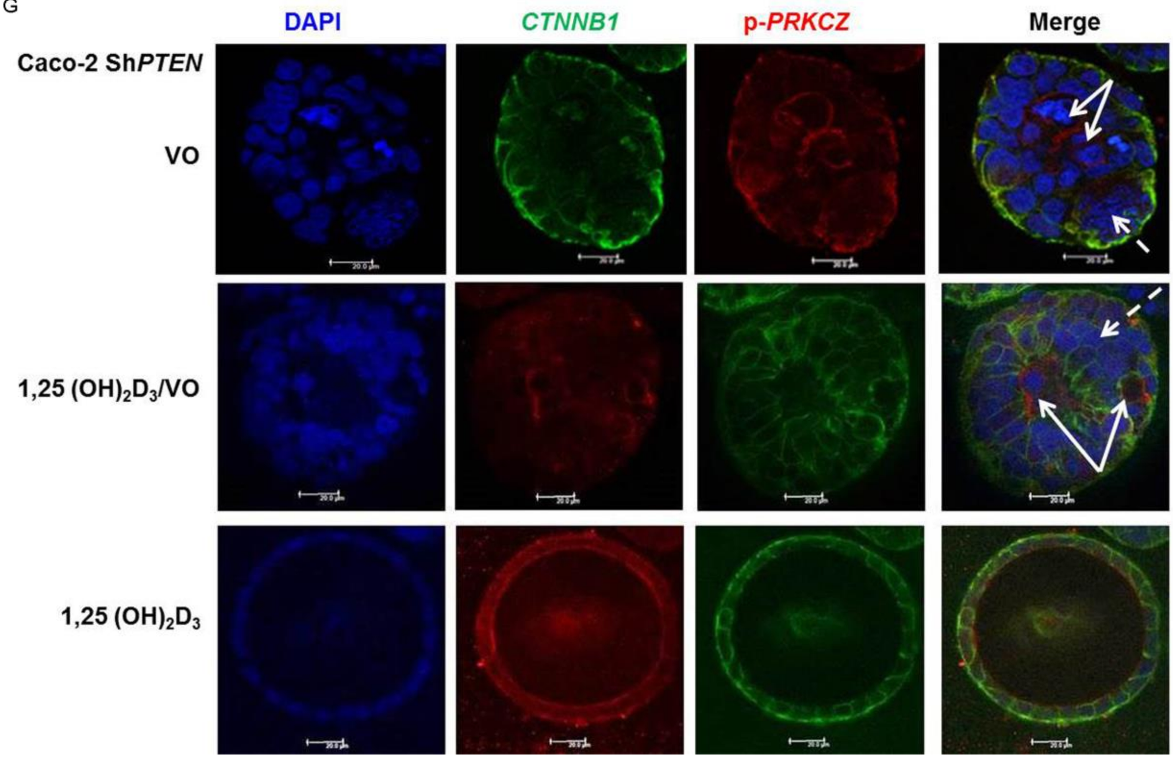

DAPI
CTNNB1
p-PRKCZ
Merge
Caco-2 ShPTEN
VO
1,25 (OH)2D3/VO
1,25 (OH)2D3

H

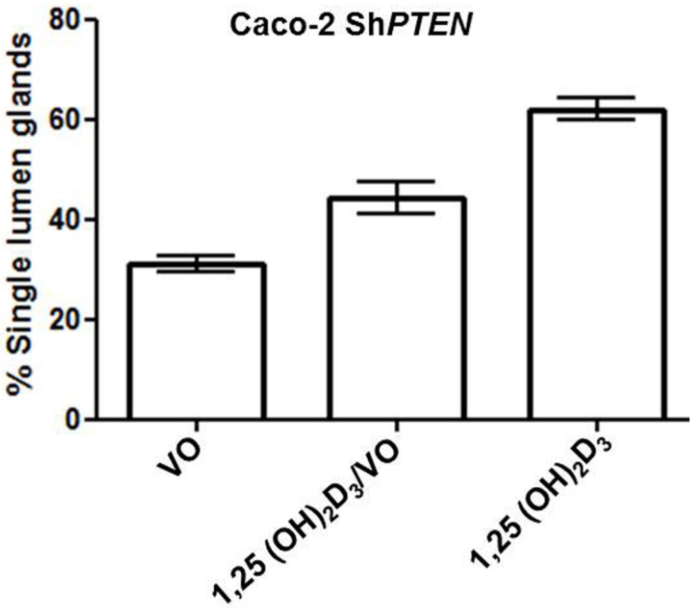

Caco-2 ShPTEN
% Single lumen glands
80
60
40
20
0
VO
1,25 (OH)2D3/VO
1,25 (OH)2D3

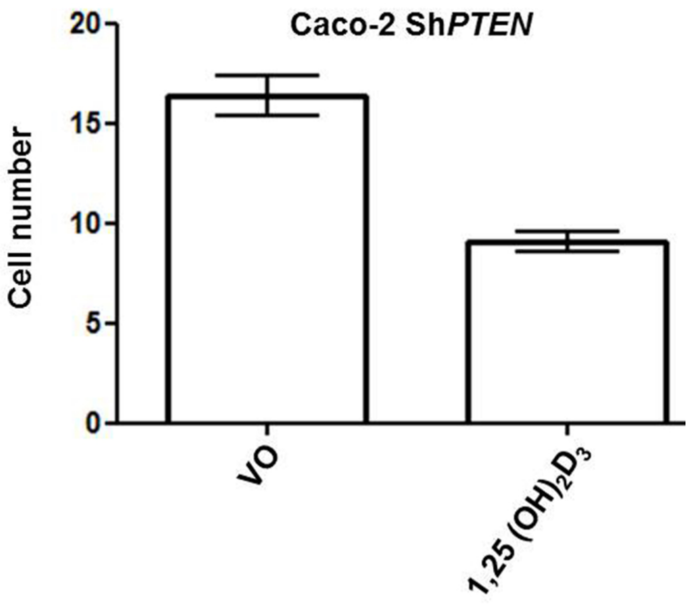

Supplementary Fig 2. A 1,25(OH)$_2$D$_3$ treatment enhances perinuclear and nuclear *VDR* immunofluorescence in Caco-2 and Caco-2 Sh*PTEN* cells. Scale bar - 20 μM. **B** Summary effects of 1,25(OH)$_2$D$_3$ treatment on *VDR* immunofluorescence. Values shown indicate total *VDR* immunofluorescence relative to VO treated Caco-2 cells (Caco-2 - 1,25(OH)$_2$D$_3$ - 3.15 ± 0.16; Caco-2 Sh*PTEN* - VO - 0. 74 ± 0.04; Caco-2 Sh*PTEN* -1,25(OH)$_2$D$_3$ - 2.9 ± 0.16; p<0.01; ANOVA). **C** Effects of 1,25(OH)$_2$D$_3$ on progressive Caco-2 Sh*PTEN* gland development. Multiple lumens and epithelial stratification are shown by solid and broad interrupted white arrows respectively in Merge images. Scale bar - 20 μM. **D** Summary 1,25(OH)$_2$D$_3$ treatment effects on single lumen formation in Caco-2 Sh*PTEN* glands. Values shown represent fold change of 1,25(OH)$_2$D$_3$ treatment (shaded bars) over VO treated control (white bars) at each temporal stage - 4 days - 1.60 ± 0.03; 8 days - 1.70 ± 0.03; 12 days - 1.95 ± 0.07 p<0.01; ANOVA). **E** Long term (20 days) 1,25(OH)$_2$D$_3$ rescue of Caco-2 Sh*PTEN* gland morphology. Top row - *VDR* (green) indicates biological responsiveness to 1,25(OH)$_2$D$_3$ *vs* DAPI (blue) nuclear imaging. Bottom row - Apical membrane identified by *SLC9A3R1* immunufluorescence (green) *vs* DAPI (blue). **F**

Summary effects of $1,25(OH)_2D_3$ treatment on single lumen formation in Caco-2 Sh*PTEN* glands at 20 days (VO - 42.33 ± 2.3%; $1,25(OH)_2D_3$ - 74.67 ± 3.480%; $p<0.01$; Student's t test). **G** Discontinuation of $1,25(OH)_2D_3$ treatment induces reversal to CM. Top panel VO for 12 days; Middle panel $1,25(OH)_2D_3$ treatment for 4 days followed by VO for 8 days; Bottom panel - $1,25(OH)_2D_3$ treatment for 12 days. DAPI, p-*PRKCZ* and *CTTNB1* used as nuclear, apical membrane and basolateral membrane markers respectively. Multiple lumens and epithelial stratification indicated by solid and broken white arrows in Merge respectively. **H** Summary effects of discontinuous or continuous $1,25(OH)_2D_3$ treatment on Caco-2Sh*PTEN* gland single lumen formation at 12 days. VO for 12 days - 31.0 ± 1.5%; $1,25(OH)_2D_3$ for 4 days followed by VO for 8 days - 44.3 ± 3.3%; $1,25(OH)_2D_3$ treatment for 12 days - 62.0 ± 2.3% ($p<0.01$;ANOVA). **I** Summary effects of continuous $1,25(OH)_2D_3$ treatment on Caco-2 Sh*PTEN* gland cellularity at 4 days of culture (VO - 16.0 ± 0.97 *vs* $1,25(OH)_2D_3$ - 9.0 ±0.56; $p<0.01$; Student's t test).

A

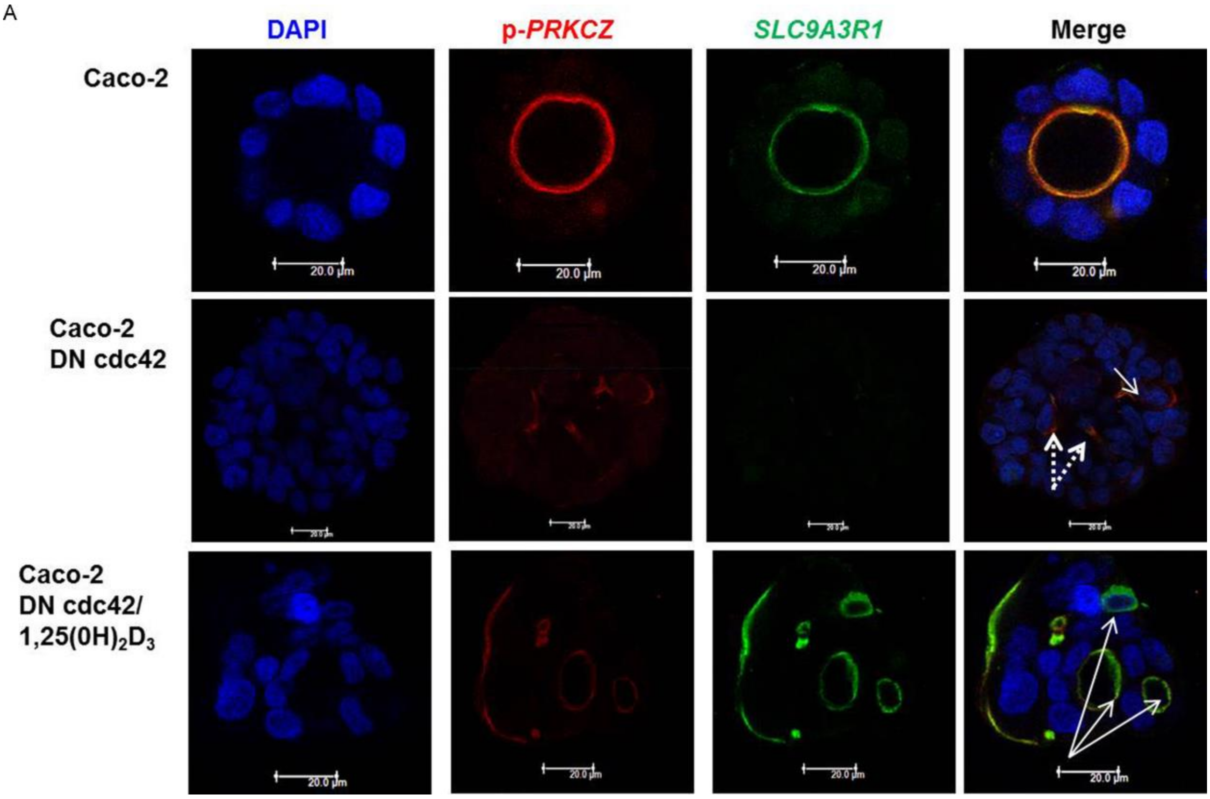

DAPI
p-PRKCZ
SLC9A3R1
Merge
Caco-2
Caco-2 DN cdc42
Caco-2 DN cdc42/ 1,25(0H)$_2$D$_3$
20.0 µm

B

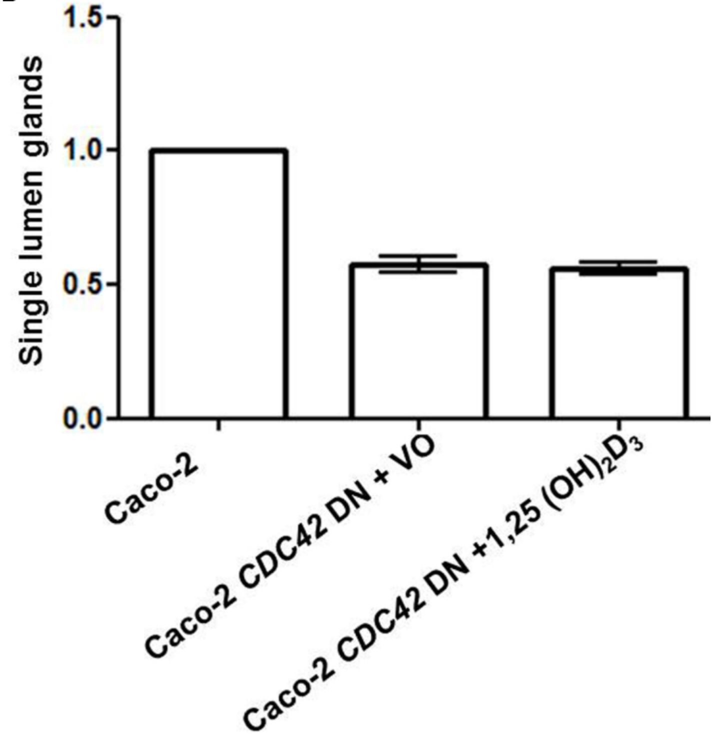

Single lumen glands
1.5
1.0
0.5
0.0
Caco-2
Caco-2 CDC42 DN + VO
Caco-2 CDC42 DN +1,25 (OH)$_2$D$_3$

C

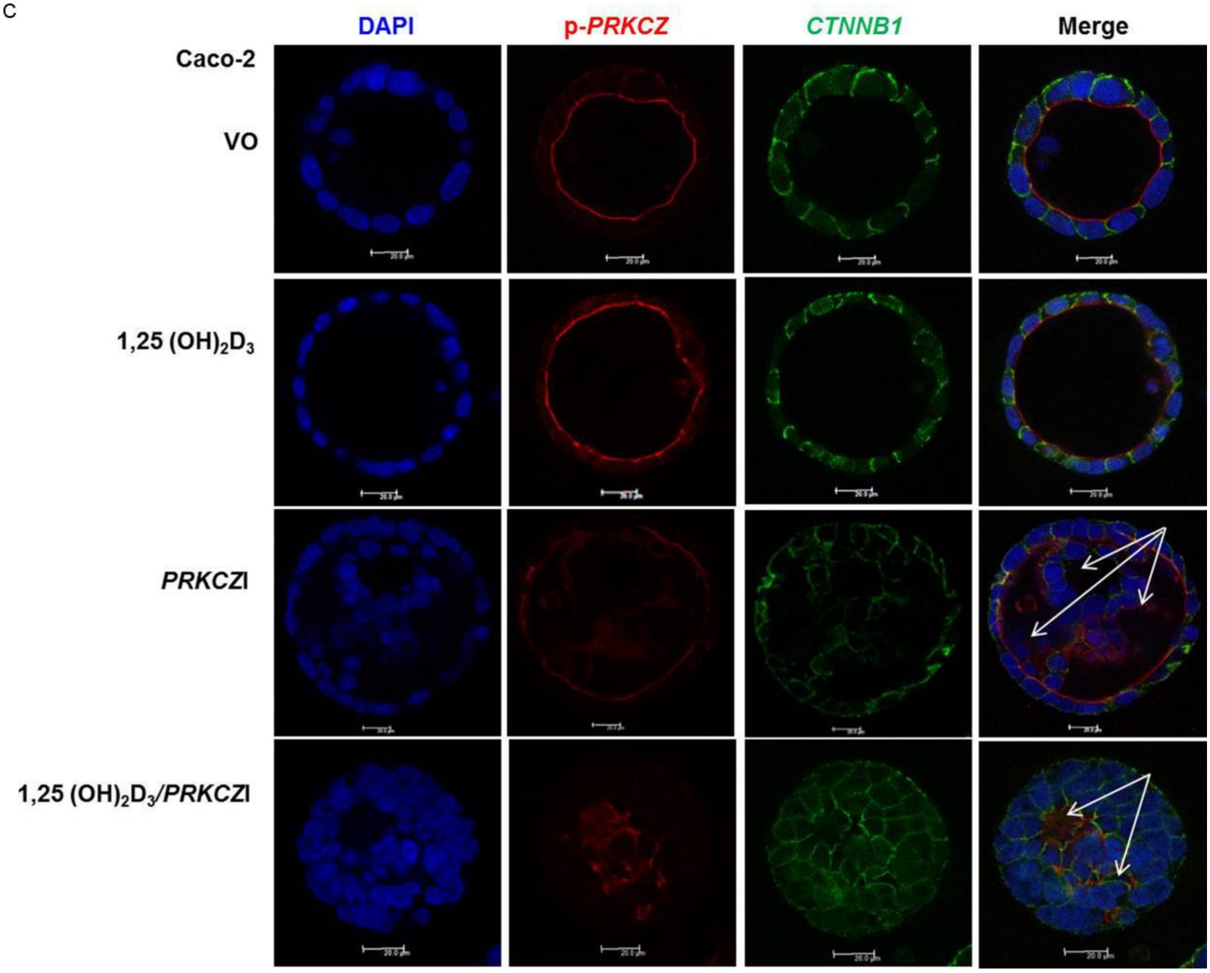
DAPI
p-PRKCZ
CTNNB1
Merge
Caco-2
VO
1,25 (OH)2D3
PRKCZI
1,25 (OH)2D3/PRKCZI

D

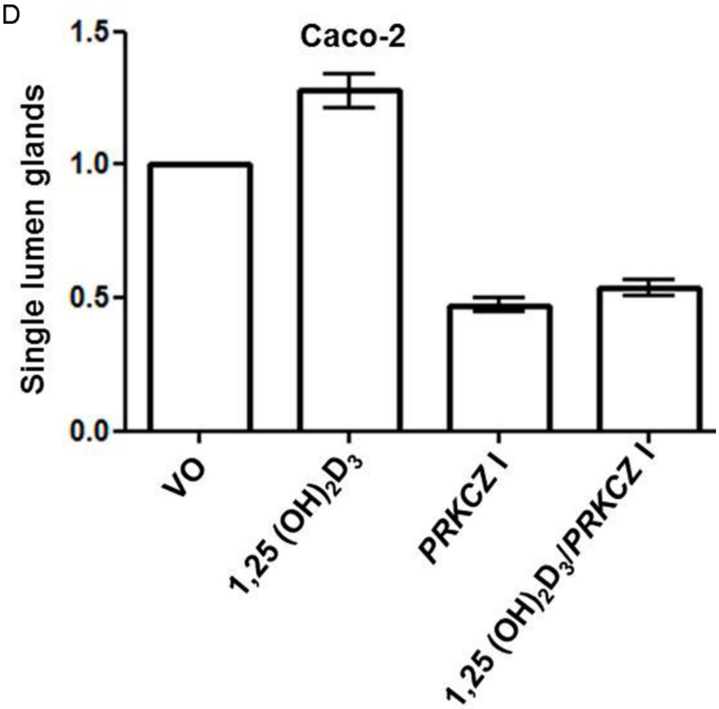
Caco-2
Single lumen glands
1.5
1.0
0.5
0.0
VO
1,25 (OH)2D3
PRKCZ I
1,25 (OH)2D3/PRKCZ I

Supplementary Fig 3. A 1,25$(OH)_2D_3$ treatment does not reverse aberrant Caco-2 gland morphology induced by dominant-negative (DN) *CDC42.* Apical p-*PRKCZ* and *SLC9A3R1* used as readouts of apical *PRKCZ* activity. Top row - Caco-2 glands transfected by empty vector (EV) and treated by treated by vehicle only (VO); second row - Caco-2 glands transfected by DN _*CDC42* and treated by VO*;* Third row Caco-2 glands transfected by DN _*CDC42* and treated by 1,25$(OH)_2D_3$. Lumen formation and ectopic AM are indicated by solid and fine interrupted white arrows respectively in Merge images. Scale bar - 20 μM. **B** Summary effects of DN_*CDC42* with or without 1,25$(OH)_2D_3$ treatment on single lumen formation in Caco-2 glands. Values shown represent fold changes over VO treated Caco-2 control at 4 days (VO treated Caco-2 DN *CDC42* - 0.57 ± 0.03; 1,25$(OH)_2D_3$ - treated Caco-2 DN *CDC42* - 0.52 ± 0.04; $p<0.01$; ANOVA). **C** 1,25$(OH)_2D_3$ treatment does not reverse aberrant Caco-2 gland morphology induced by *PRKCZ*I treatment. DAPI, p-*PRKCZ* and *CTNNB1* used as markers of nuclear DNA, apical and basolateral membranes respectively. Top row - VO; second row - 1,25$(OH)_2D_3$; third row - *PRKCZ*I; fourth row - 1,25$(OH)_2D_3$ /*PRKCZ* I in combination. Multilumen formation indicated by solid white arrows in Merge. Scale bar - 20 μM. **D** Summary effects of *PRKCZ*I with or without 1,25$(OH)_2D_3$ treatment on single lumen formation in Caco-2 glands. Values shown represent fold change over VO treated Caco-2 control (1,25$(OH)_2D_3$ - 1.30 ± 0.06; *PRKCZ* I - 0.50 ± 0.03; 1,25$(OH)_2D_3$ + *PRKCZ* I - 0.54 ± 0.03 $p<0.01$; ANOVA).

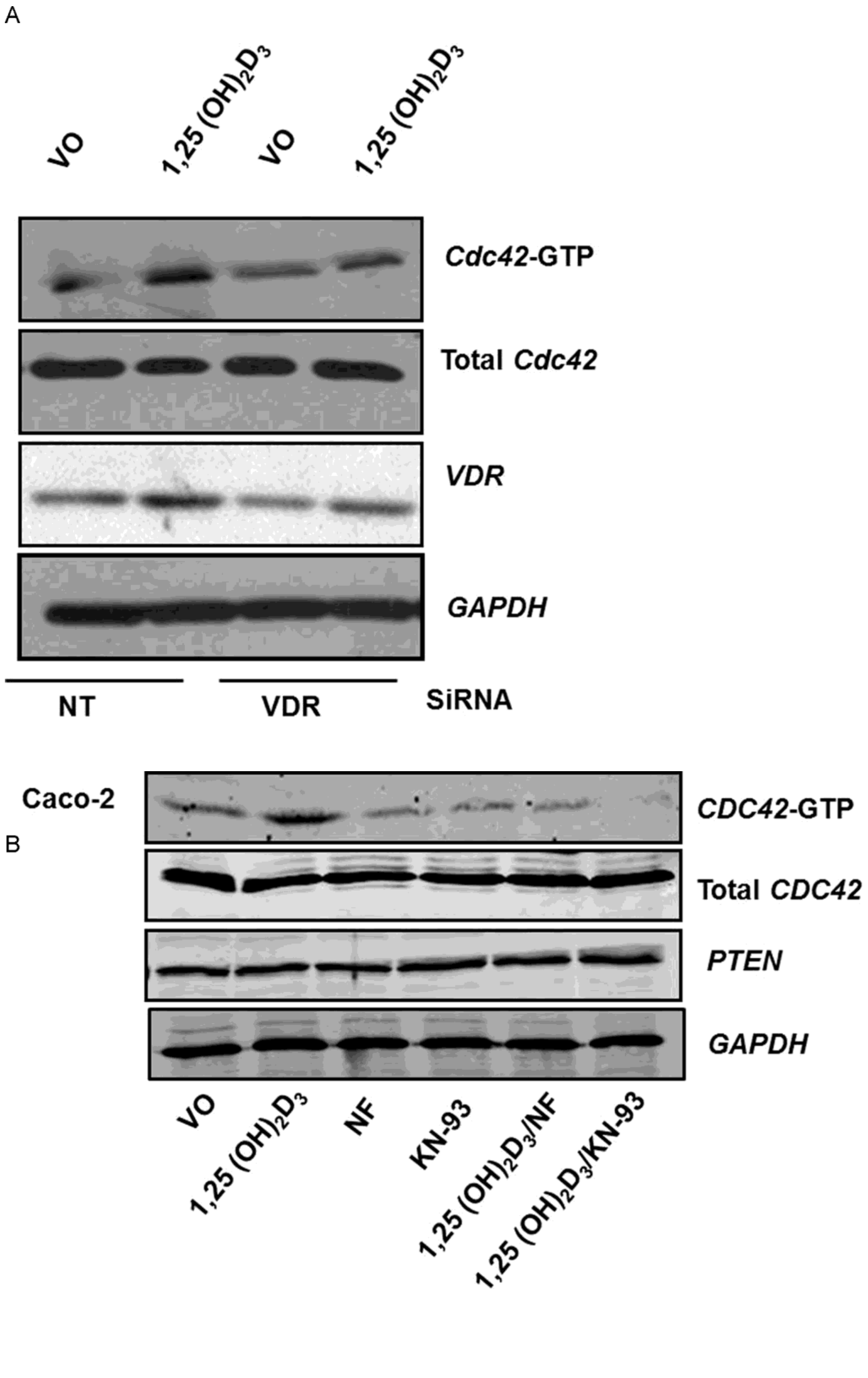
A
VO
1,25 (OH)2D3
VO
1,25 (OH)2D3
Cdc42-GTP
Total Cdc42
VDR
GAPDH
NT
VDR
SiRNA
Caco-2
B
CDC42-GTP
Total CDC42
PTEN
GAPDH
VO
1,25 (OH)2D3
NF
KN-93
1,25 (OH)2D3/NF
1,25 (OH)2D3/KN-93

C

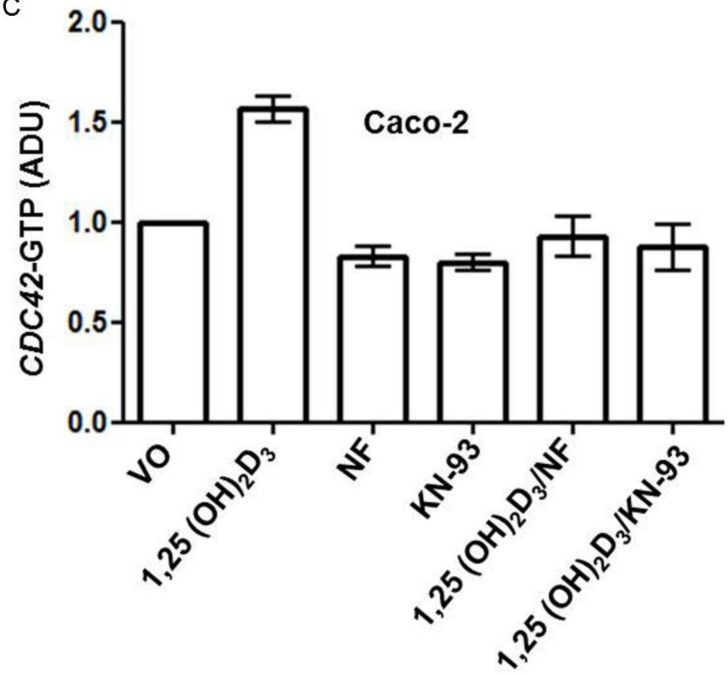

Caco-2
$CDC42$-GTP (ADU)
2.0
1.5
1.0
0.5
0.0
VO
1,25 $(OH)_2D_3$
NF
KN-93
1,25 $(OH)_2D_3$/NF
1,25 $(OH)_2D_3$/KN-93

D

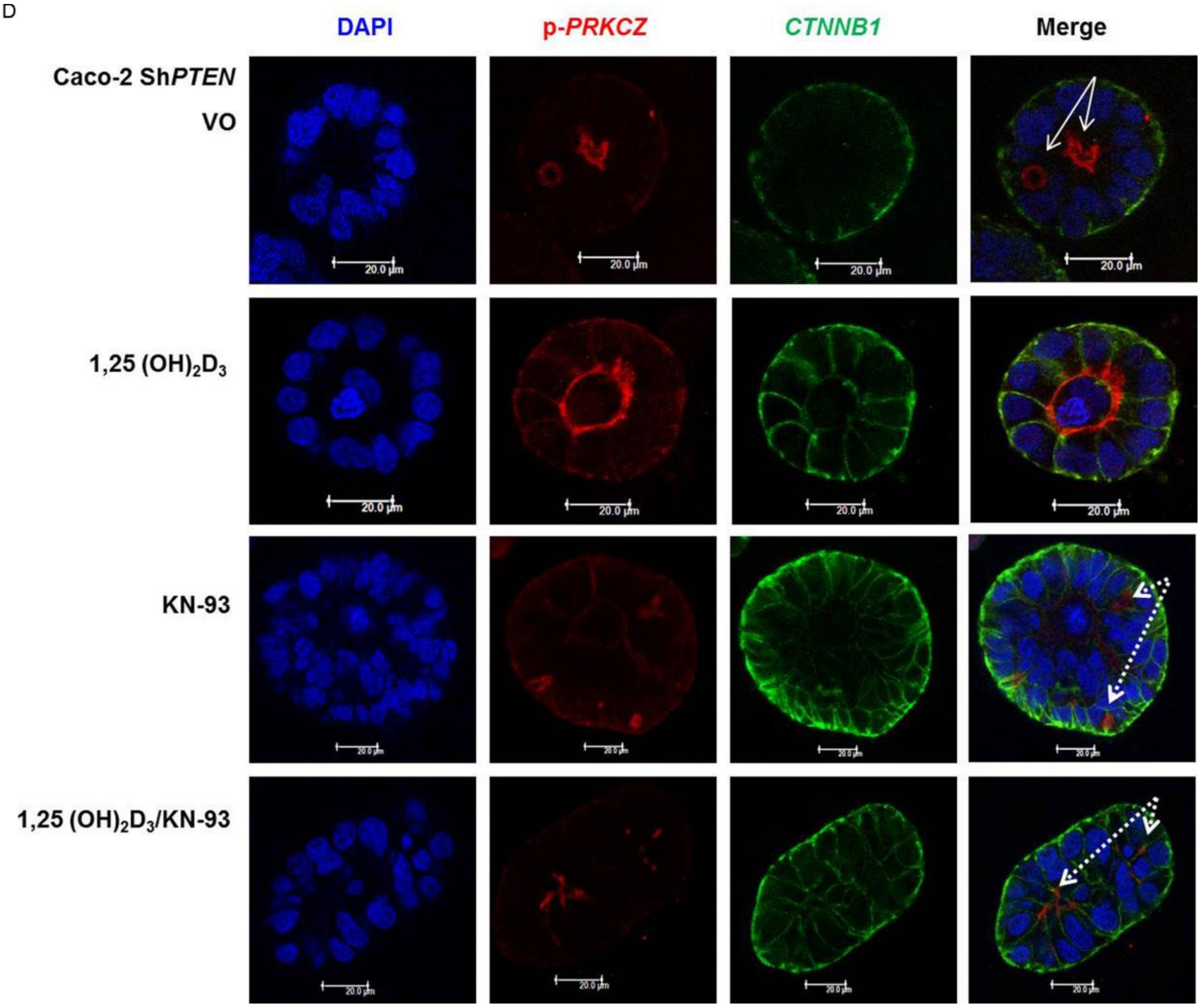

DAPI
p-*PRKCZ*
CTNNB1
Merge
Caco-2 Sh*PTEN*
VO
1,25 $(OH)_2D_3$
KN-93
1,25 $(OH)_2D_3$/KN-93
20.0 µm

E

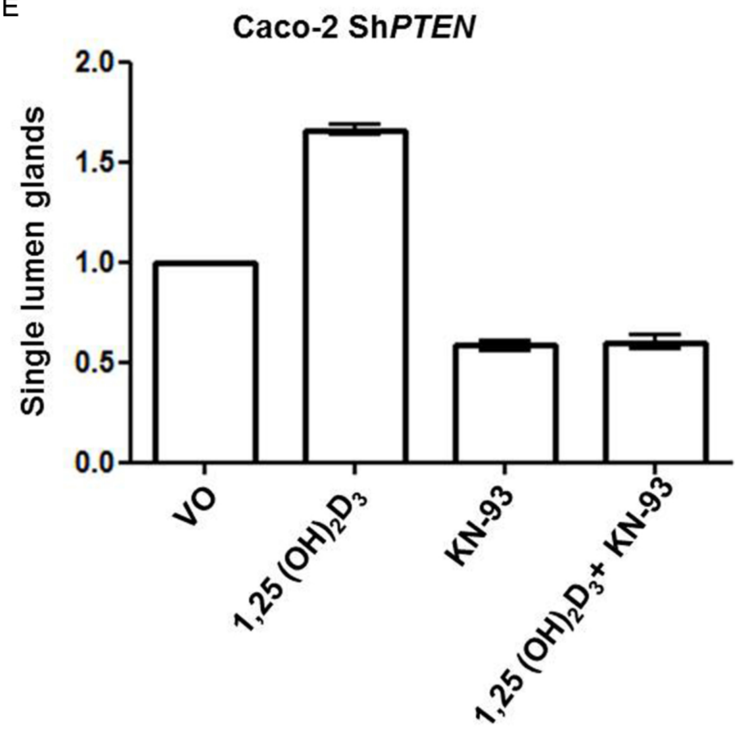

Supplementary Fig 4. A Knockdown of *VDR* impedes 1,25(OH)$_2$D$_3$ activation of *CDC42.* SiRNA suppression of *VDR* inhibits 1,25(OH)$_2$D$_3$ activation of *CDC42 vs* Non-targeting (NT) SiRNA. **B** Ca $^{2+}$ blockade suppresses 1,25(OH)$_2$D$_3$ activation of *CDC42.* Expression of *CDC42*-GTP and *PTEN* after treatment of Caco-2 cells by VO, 1,25(OH)$_2$D$_3$, nifedipine (NF), 1,25(OH)$_2$D$_3$ + NF, KN-93 or 1,25(OH)$_2$D$_3$ + KN-93. **C** Summary effects of Ca^{2+} blockade on 1,25(OH)$_2$D$_3$ activation of *CDC42.* Densitometry assays of *CDC42*-GTP after treatment. Values shown are densitometry fold expression differences against VO control treatment (1,25(OH)$_2$D$_3$ - 1.56 ± 0.06; NF - 0.82 ± 0.05; KN-93 - 0.80 ± 0.04; 1,25(OH)$_2$D$_3$ + NF - 0.93 ± 0.10; 1,25(OH)$_2$D$_3$ + KN-93 - 0.75 ± 0.12; p <0.02 for 1,25(OH)$_2$D$_3$ *vs* VO; ANOVA). **D** CaM-KII blockade suppresses1,25(OH)$_2$D$_3$ rescue of multicellular morphology. Caco-2 Sh*PTEN* gland morphogenesis after treatment by VO (top row), 1,25(OH)$_2$D$_3$ (second row) , KN-93 (third row) or 1,25(OH)$_2$D$_3$/KN-93 in combination (fourth row). Lumens and ectopic AM indicated by solid and fine interrupted white arrows

respectively in Merge images. Scale bar - 20 μM. **E** Summary effects of CaM-KII blockade on 1,25$(OH)_2D_3$ rescue of single lumen formation in Caco-2 Sh*PTEN* glands. Values are expressed as fold changes over VO treated control (1,25$(OH)_2D_3$ - 1.66 ± 0.04; KN-93 - 0.58 ± 0.04; 1,25$(OH)_2D_3$/KN-93 - 0.60 ± 0.06; $p<0.01$ ANOVA).

A

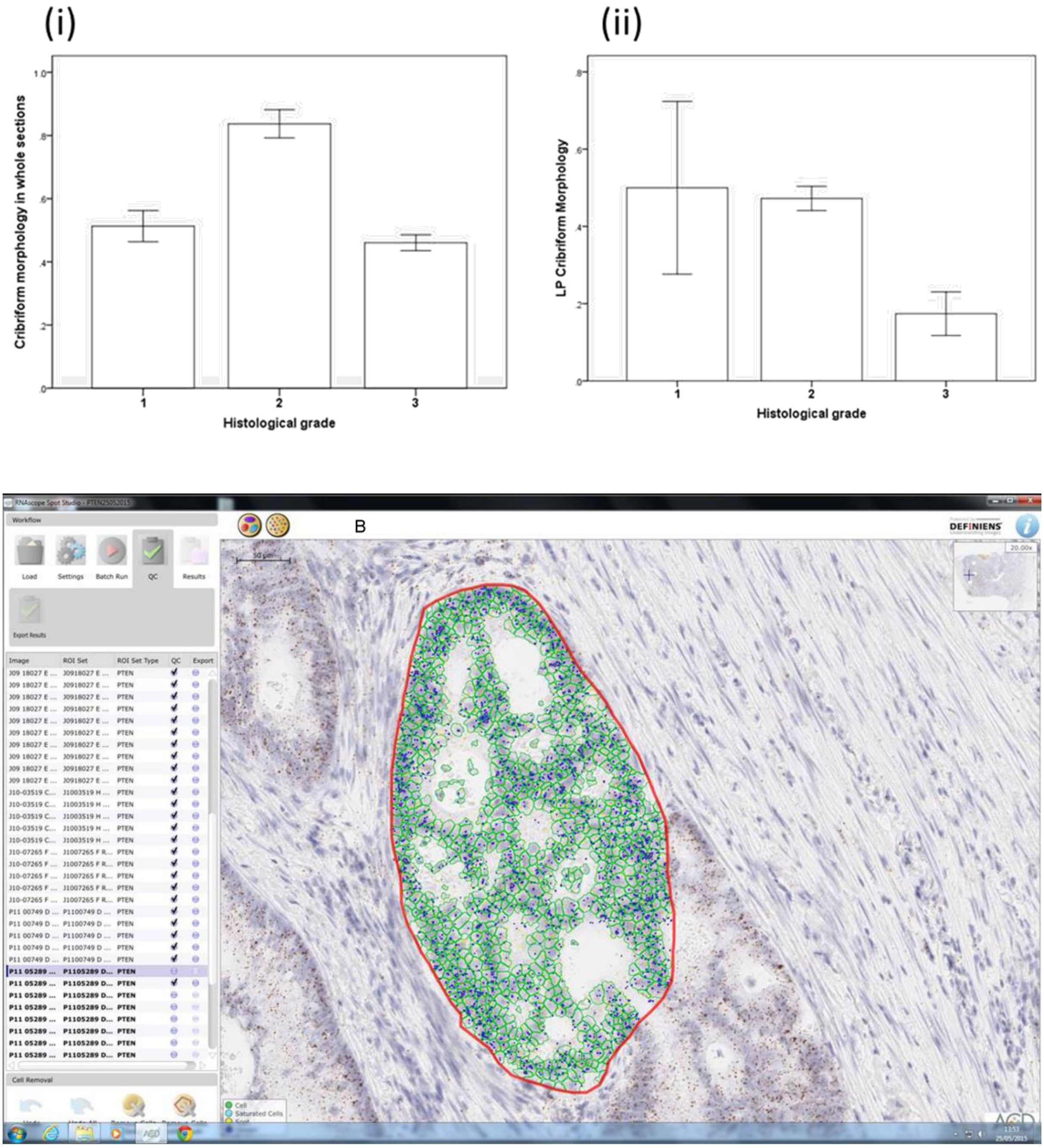
(i)
Cribriform morphology in whole sections
1.0
.8
.6
.4
.2
.0
1
2
3
Histological grade
(ii)
LP Cribriform Morphology
.8
.6
.4
.2
.0
1
2
3
Histological grade
B
Workflow
Load
Settings
Batch Run
QC
Results
Export Results
Image
ROI Set
ROI Set Type
QC
Export
PTEN
Cell Removal
Cell
Saturated Cells
DEFINIENS
20.00x
50 µm
25/05/2015

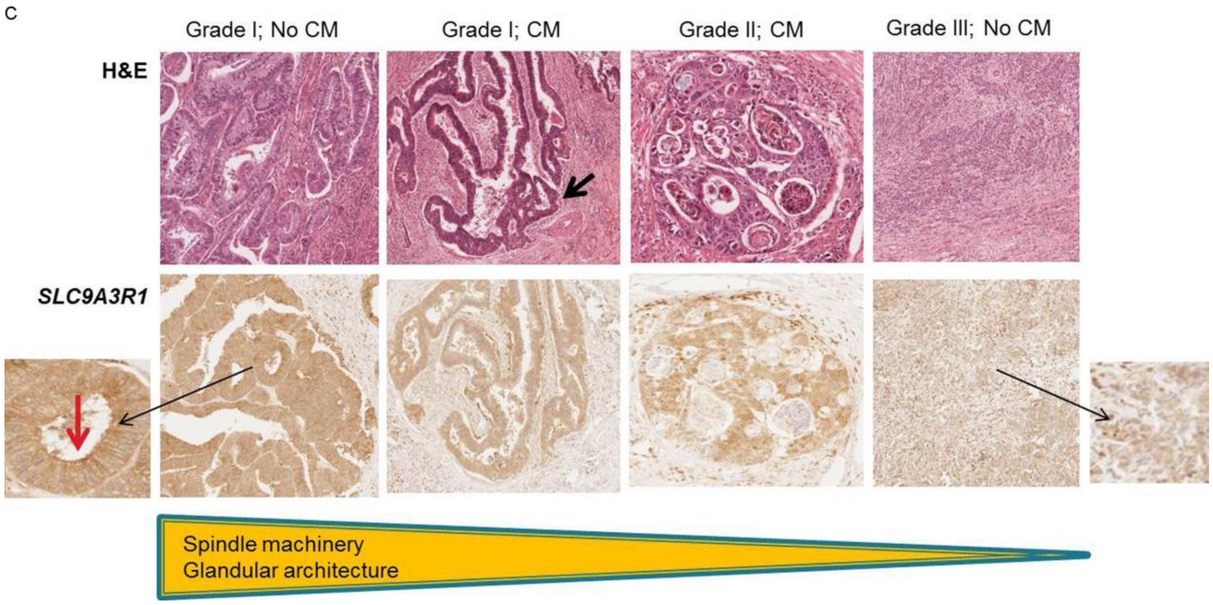

Supplementary Fig 6. A Cribriform morphology and colorectal cancer grade in 2 study cohorts. CM was assessed throughout 40 fields of whole tumor sections in cohort (i) and at low power microscopy only in cohort (ii). Mean CM values for grades I-III CRCs in cohort (i) were 0.51 ± 0.05; 0.84 ± 0.45; 0.46 ± 0.25; p<0.01;ANOVA and cohort (ii) 0.50 ± 0.22; 0.47 ± 0.03; 0.17 ± 0.06; p<0.01). **B** *PTEN* RNAScope assays in human CRC. *PTEN* RNA situ hybridization was quantified as the number of positive signal foci (spots) per cell. Regions of interest (ROIs) were defined by manual annotation in each tumor. The example shows selection of cribriform morphology. Single-cell automated quantitation of RNA transcripts was conducted using the RNAScope StudioSpot software suite (Advanced Cell Diagnostics [ACD], CA 94545, USA). **C** Decline of apical *SLC9A3R1* intensity associates with CRC dedifferentiation. In human colon, greatest apical *SLC9A3R1* intensity is found in normal mucosa. In cancers, grade I tumors show high apical *SLC9A3R1* immunolocalization (arrow in high power insert). A trajectory of gland destabilization between grade I and III CRCs is accompanied by a decline of apical *SLC9A3R1* intensity.
